# Bulbar Microcircuit Model Predicts Connectivity and Roles of Interneurons in Odor Coding

**DOI:** 10.1371/journal.pone.0098045

**Published:** 2015-05-05

**Authors:** Aditya Gilra, Upinder S. Bhalla

**Affiliations:** National Centre for Biological Sciences (NCBS), Tata Institute of Fundamental Research (TIFR), Bangalore, 560065, India; MRC-National Institute for Medical Research, UNITED KINGDOM

## Abstract

Stimulus encoding by primary sensory brain areas provides a data-rich context for understanding their circuit mechanisms. The vertebrate olfactory bulb is an input area having unusual two-layer dendro-dendritic connections whose roles in odor coding are unclear. To clarify these roles, we built a detailed compartmental model of the rat olfactory bulb that synthesizes a much wider range of experimental observations on bulbar physiology and response dynamics than has hitherto been modeled. We predict that superficial-layer inhibitory interneurons (periglomerular cells) linearize the input-output transformation of the principal neurons (mitral cells), unlike previous models of contrast enhancement. The linearization is required to replicate observed linear summation of mitral odor responses. Further, in our model, action-potentials back-propagate along lateral dendrites of mitral cells and activate deep-layer inhibitory interneurons (granule cells). Using this, we propose sparse, long-range inhibition between mitral cells, mediated by granule cells, to explain how the respiratory phases of odor responses of sister mitral cells can be sometimes decorrelated as observed, despite receiving similar receptor input. We also rule out some alternative mechanisms. In our mechanism, we predict that a few distant mitral cells receiving input from different receptors, inhibit sister mitral cells differentially, by activating disjoint subsets of granule cells. This differential inhibition is strong enough to decorrelate their firing rate phases, and not merely modulate their spike timing. Thus our well-constrained model suggests novel computational roles for the two most numerous classes of interneurons in the bulb.

## Introduction

**Fig 1:**
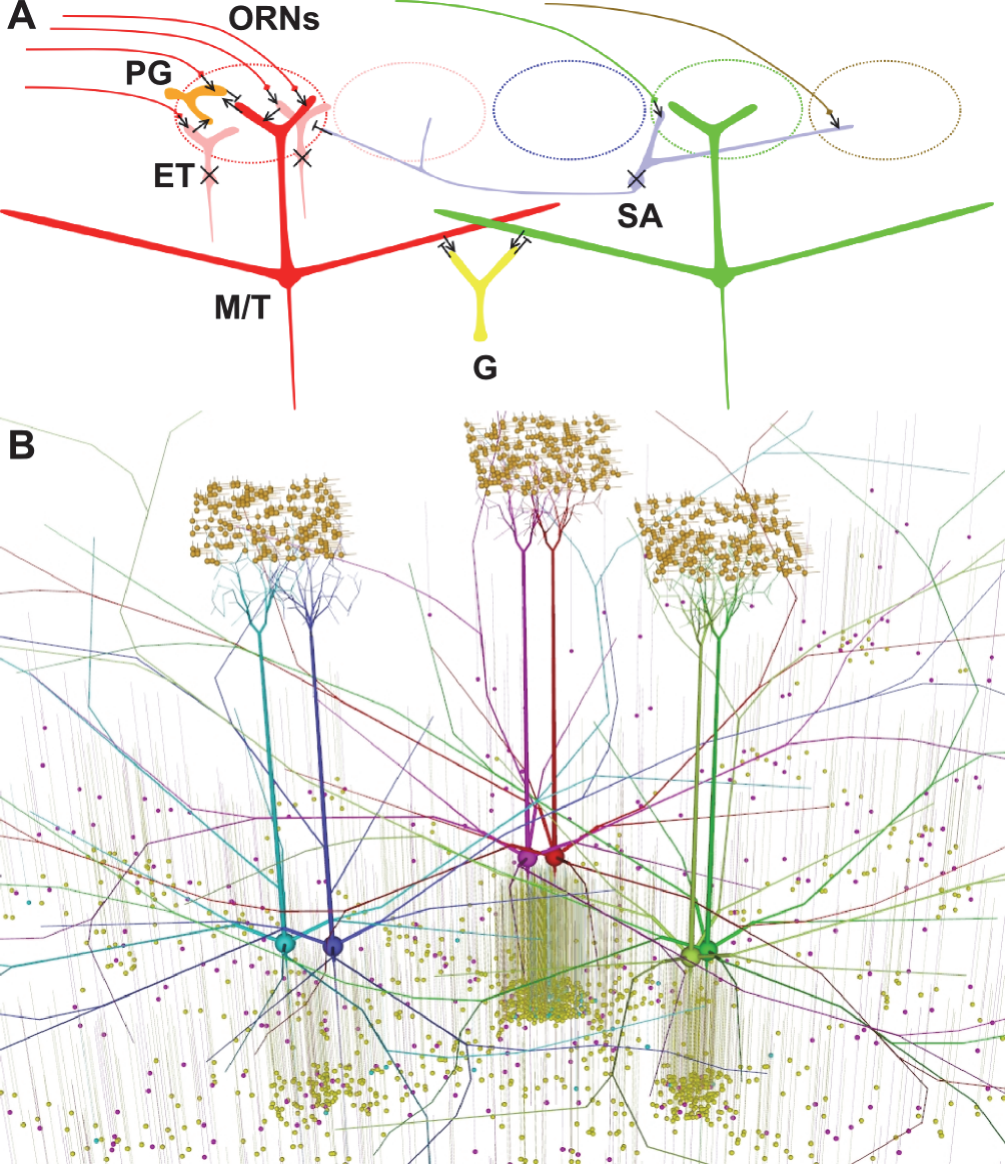
Model connectivity. A. Synaptic schematic: Each glomerulus (dotted ellipse) receives input from olfactory receptor neurons (ORNs) expressing a single type of receptor out of many (different colors). Mitral/tufted (M/T) cells take excitatory input onto their dendritic tufts within one glomerulus, directly from ORNs (and via ET cells). ET cells have not been modeled (crossed out) and their input to M/T and PG cells is considered folded into the ORN input. Periglomerular (PG) cells are excited by ORNs (and via ET cells), and in turn inhibit M/T cells within the same glomerulus, thus causing feed-forward inhibition. PG cells also get excitation from M/T cells at reciprocal synapses, thus mediating recurrent inhibition. Further, M/T cells form reciprocal synapses with granule cells on their soma, primary and lateral dendrites, where they excite granule (G) cells which cause recurrent and lateral inhibition. Short-axon (SA) cells have not been modeled (crossed out). B. Visualization of *default* model having 3 glomeruli each with 2 sister mitral cells, and connecting interneurons. Singly connected granule cells are shown in purple. The jointly and multiply connected (shared) granule cells are shown in yellow and cyan respectively. PG cells are shown in orange. Synaptic connections are not shown, but granule and PG cells connect to nearby mitral dendrites, within their small dendritic extents.

Primary sensory encoding provides a particularly direct framework for studying input-output computations in the brain. In sensory systems like vision, there is a direct topological mapping of the two-dimensional visual field onto a two-dimensional neuronal substrate. In contrast [1], olfactory stimuli occupy a high-dimensional space [2,3] and are represented by patterns of spatio-temporal activation of glomeruli on the two-dimensional surface of the olfactory bulb (OB) [4,5]. These are further transformed into the spiking patterns of bulbar principal neurons i.e. the mitral/tufted (M/T) cells, via the distinctive dual-layer dendro-dendritic circuitry (Fig 1A) of the olfactory bulb [6,1].

There is a distinguished history of models that explore the implications of this dendro-dendritic circuitry [7,8]. Intra-glomerular dendro-dendritic inhibition by periglomerular cells performs non-topographic contrast enhancement in some models [9,10]. In others, dendro-dendritic inhibition by granule cells synchronizes and modulates mitral spike times [11–15], and spatio-temporally sculpts odor responses [15]. However, very few models span the range from circuit-level physiology to replicating temporal and cross-neuron odor coding features from multiple *in vivo* experiments. Thus, substantial gaps remain in our understanding of cellular, dendro-dendritic, and network mechanisms for odor coding in the olfactory bulb.

Here, we report a detailed model of micro-circuits in the rat olfactory bulb to understand and predict the circuit mechanisms that account for its major odor coding properties. Our model has been constrained hierarchically, using multiple single-cell and coupled-cell recordings, both *in vitro* and *in vivo*, chosen to probe different levels of the circuitry. As a strong test of the model, we match its output against multiple *in vivo* experimental findings on linear coding [16,17] and decorrelation [18] that provide direct measurements of the input-output transformations occurring in the rodent olfactory bulb. We predict that contrary to models of contrast enhancement that propose non-linear input-output transformations [9,10], the glomerular tuft microcircuit plays a key role in linearization. We further predict that there are sparse long-range outputs, mediated by secondary dendrites and granule cell columns, which are responsible for decorrelating respiratory phases, rather than merely modulating spike timing.

## Results

We used multi-scale compartmental modeling to first match cell- and synapse-level observations of bulbar anatomy and physiology, and then to build a microcircuit network model to replicate *in vitro* coupled-cell recordings and *in vivo* experiments on odor responses. We then tested the model on responses to various patterned odor stimuli comprising single and binary odors. We finally performed a series of simulated lesion and circuit reconfiguration experiments to understand the mechanistic basis for linear summation of odorant responses, and decorrelation of phases of sister mitral cell responses.

### Model overview

In order to span the range from cellular physiology to single- and cross-glomerular mitral cell coding, our model included simulated olfactory receptor neuron (ORN) input, periglomerular (PG) cells, mitral/tufted (henceforth termed mitral) cells, and granule cells. To study the odor responses of single or coupled mitral cells, we organized our model into a central odor-responsive glomerulus with two representative sister mitral cells, and 0 to 6 odor-responsive lateral glomeruli, each with two mitral cells, that could strongly influence via interneurons the two central sister mitral cells of interest. These mitral cells were coupled with physiological numbers of PG and granule cells to complete the dendro-dendritic microcircuits.

We tried various connectivities: *random*, *directed*, and *default* in our simulations as introduced in the Results sub-section ‘Lateral dendrites deliver rather than receive inhibition’. Our final *default* model (Fig 1B) had: (a) 3 glomeruli, each with 2 mitral cells and 1000 PG cells; (b) ~1200 granule cells shared between 2 or more of the 6 mitral cells; and (c) ~95 granule cells (each representing 100 cells—see Materials and Methods) connected singly to each of the 6 mitral cells, i.e. ~570 singly-connected granule cells. We provided background Poisson spikes to all granule cells as a proxy for input from the large number of mitral cells that were not modeled. Final parameter values and rationale are summarized in Tables 1 and 2, and detailed in Materials and Methods. As notation, we use A → B to denote excitatory synapse from cell A to cell B; A ─┤B to denote inhibitory synapse from cell A to cell B; and A├→ B to denote a reciprocal synapse where cell A excites cell B while cell B inhibits cell A; and similarly B ←┤A. Although we constrained the model in hierarchical stages (Materials and Methods), only the results for the fully constrained *default* model are reported below.

**Table 1:** Experimental and model cell numbers, along with incoming synaptic numbers, strengths, and time constants.

Cell	Number	Synapse	Number of synapses per cell	Experiment: PSP / PSC amplitude, peak / fall times	Modeling strategy	Model: max conductance & time constants; Simulated PSP amplitude & peak / fall times
**ORN**	10^4^ / glomerulus				As Poisson spikes	
**PG**	70–85% of juxtaglomerular (JG) cells (1500–2000 [20]) [21] i.e. ~1000 / glomerulus	ORN→PG	~50 spines (estimated from 25 in mice [22]).	EPSP 3 mV [23,24], τ_f_ ~ 5 ms [24]	1000 PG / glomerulus; 50 ORN→PG synapses per PG.	g_max_ = 0.45 nS for plateauing and 1.25 nS for low-threshold spiking PG cell, τ_1_ = 1 ms, τ_2_ = 1 ms (same for both synapses); simulated EPSP: ~7-8 mV, τ_p_ ~ 2 ms, τ_f_ ~ 5 ms.
		M/T→PG or ET→PG	Similar to above.	EPSP 6–10 mV [24,25], τ_f_ ~ 5 ms [24]	25 M→PG per PG.	Same as above.
**ET**	10% of JG cells (M.T. Shipley, email, 2010) [26,27]. Hence ~150–200 / glomerulus	ORN→ET; SA─┤ET			Absorbed into ORN Poisson spikes.	
**SA**	15–20% of JG cells are ET / SA [26] (mice). 1:1 with M/T [27]. So, ~100 / glomerulus.				Not much inter-glomerular inhibition *in vivo* [28], so ignored.	
**M/T**	25 M / 50 T per glomerulus [27]	ORN→M	460–1500 [29]	Conflicting EPSP amplitudes: ~3 mV [30], to ~0.1 mV [29], τ_p_ = 6 ms, τ_f_ = 12 ms [30].	2 mitral cells / glomerulus; 400 ORN→M on mitral tuft.	g_max_ = 6 nS, τ_1_ = 1 ms, τ_2_ = 1 ms; simulated EPSP: ~1 mV, τ_p_ ~ 7 ms, τ_f_ ~ 80 ms (Large time constant of our mitral cell model caused long EPSPs)
		G ─┤M	10^4^ [11,31]	IPSC amplitude decays with distance [32]. IPSCs have τ_p_ ~ 5 ms, τ_f_ ~ 30 ms [33]; spontaneous IPSPs are not well resolved, but may have similar τ_p_ = 5 ms, τ_f_ = 30 ms [34,35].	10^4^ G ─┤M on mitral soma, apical and lateral dendrites.	proximal g_max_ = 1 nS but 4× if *‘s*uper-inhibitory’ in *default* network (1.5 nS in *random* / *directed* network as it had no ‘super-inhibitory’ synapses), τ_1_ = 1 ms, τ_2_ = 20 ms; simulated IPSP: ~ –0.9mV (proximal), τ_p_ ~ 26 ms, τ_f_ ~ 115 ms; (long IPSPs as above. We verified that reducing τ_2_ to 1 ms to get short IPSPs did not affect our results qualitatively, since g_max_ was set by activity-dependent inhibition, and its value had to increase (to 12 nS) to compensate. In any case, the composite IPSP due to multiple granule cells has τ_f_ > 200 ms [36,37].)
		PG ─┤M	~100 (PG spines are connected to both M/T and ET i.e. ~250 cells)	IPSCs are similar to above G ─┤M synapse [38].	100 PG ─┤M on mitral tuft,	g_max_ = 1 nS, τ_1_ = 1 ms, τ_2_ = 20 ms; simulated IPSP: ~ –0.2 mV, τ_p_ ~ 28 ms, τ_f_ ~ 117 ms; (as above, we verified that setting τ_2_ = 1 ms with g_max_ = 30 nS did not change our results qualitatively.)
**G**	50 to 100 G per M/T [27]	M→G	100 spines [27]. Assume each spine has a reciprocal synapse.	EPSP ~3.5 mV *in vivo* [39]; τ-s from sources [39,40,33]; Mg-block voltage-dependence and NMDA to AMPA ratio from experiment [33].	2500 G-s per glomerulus: shared G-s were retained 1:1; but non-shared were aggregated 100:1; unconnected were pruned.	AMPA: g_max_ = 0.2 nS, τ_1_ = 1 ms, τ_2_ = 4 ms; NMDA: g_max_ = 0.26 × AMPA g_max_, τ_1_ = 25 ms, τ_2_ = 200 ms; (3× for distal ‘super-inhibitory’ synapses); simulated EPSP: ~2 mV, τ_p_ ~ 13 ms, τ_f_ ~ 50 ms.

τ_p_ is the time to peak, and τ_f_ the time to fall (to 20% of peak) in the relevant cell. All synaptic conductances were modeled as dual exponential

$$g\left(t,\tau_{1},\tau_{2}\right)=Ag_{max}\left(\text{exp}\left(-t/\tau_{1}\right)-\text{exp}\left(-t/\tau_{2}\right)\right)/\left(\tau_{1}-\tau_{2}\right)$$

for *t* > 0, with time constants τ_1_ and τ_2_, and *A* to normalize the peak to *g*_*max*_. ORN input had zero delay, excitatory synapses had 1.8 ms delay, and inhibitory synapses had 0.6 ms delay from pre-synaptic spike to post-synaptic event. Reversal potential was 0 mV for excitatory, and -78 mV for inhibitory [19] synapses.

**Table 2:** Hierarchical construction of model by replicating and predicting network properties at each stage.

Sl.	Phenomenon replicated	Network adjustments	Replication / explanation / prediction
**1.**	Mean firing rate of odor responses of mitral cells in anesthetized, freely-breathing rats and mice is ~12 Hz (calculated from experimental data [16,18]). Mean mitral response for air is ~half that for odor. Mean firing rate of ORNs in mice/rat for air is ~1–2 Hz [41,42], for odor not so well-characterized [42,43].	We adjusted ORN→M strength to get mean odor / air mitral rate in freely-breathing simulations of ~14 Hz / ~8 Hz with input of experimentally typical receptor firing ranges for odor (1% saturated vapor) / air (see Stimulus protocols sub-section).	
**f2**	PG cells respond to odor in anesthetized freely-breathing rats [44,45].	We adjusted ORN→PG and M→PG strengths so that PG cells fire with odor [44,45]. We set PG ─┤M strength equal to G ─┤M (set below) (IPSCs in sub-glomerular slice and intact slice are similar in height [38]).	Mitral output vs receptor input plots with and without PG inhibition in Fig 2I, which is consistent with strong effect of PG cells [46].
**3**	Action potential is generated in mitral tuft for weak nerve shock and in mitral soma for strong nerve shock *in vitro* [47].	To the Bhalla and Bower mitral cell model [48], we added a special Na channel [49] for the initial segment, following a model for this experiment [49].	Replication in Fig 2A–2C.
**4**	Activity dependent inhibition between two mitral cells ~50 μm apart, observed in 15 of 29 mitral cell pairs probed *in vitro* [37].	We adjusted the M→G strength so that only when both mitral cells fire at intermediate rates, the shared granule cells spike. This set the point of onset of inhibition in Fig 3C. We adjusted G ─┤M strength to obtain observed mean inhibition.	Replication in Fig 3A–3D.
**5**	G—┤M conductance density drops along the primary and secondary dendrites exponentially [32].	We set the observed decay in the model as shown in Fig 4D(ii-iii).	1. Replicates spike travelling along the lateral dendrites even with local inhibition en route [32], shown in Fig 2E. 2. Implies asymmetric inhibition, in which lateral dendrites are transmitters, not receivers of inhibition, as in Fig 4F.
**6**	Linearity of responses in time and between odors [17]	PG ─┤M should not be so strong as to quench mitral firing and make the Fig 2I non-linear.	Replication in Fig 5. Dissection of the contribution of various inhibitory influences and input non-linearity to linear coding in Fig 6 and S7 Fig Prediction that PG cells control linearity more than granule cells in Fig 6.
**7**	Phase and delta-rate decorrelation of sister mitral cells [18]	Strengthened and created extra M→G and G ─┤M between central sister and a non-sister odor responsive mitral cell. Similarly between other central sister and non-sister mitral cell of a third glomerulus. Proximal strengths are set by activity dependent inhibition. Also self-inhibition should not be too strong. This enforces M→G to be stronger away from the soma, to deliver strong inhibition, yet not self-inhibit.	Strengthened synapses in the *default* network compared to *directed* network are shown in Fig 4D(ii-v). Replication in Fig 7. Testing alternative network configurations in Fig 8. Prediction that strong, directed, differential and sparse inhibitory connections between non-sister mitral cells are needed for phase-decorrelation.

### Model cells match experimental electrophysiology

**Fig 2:**
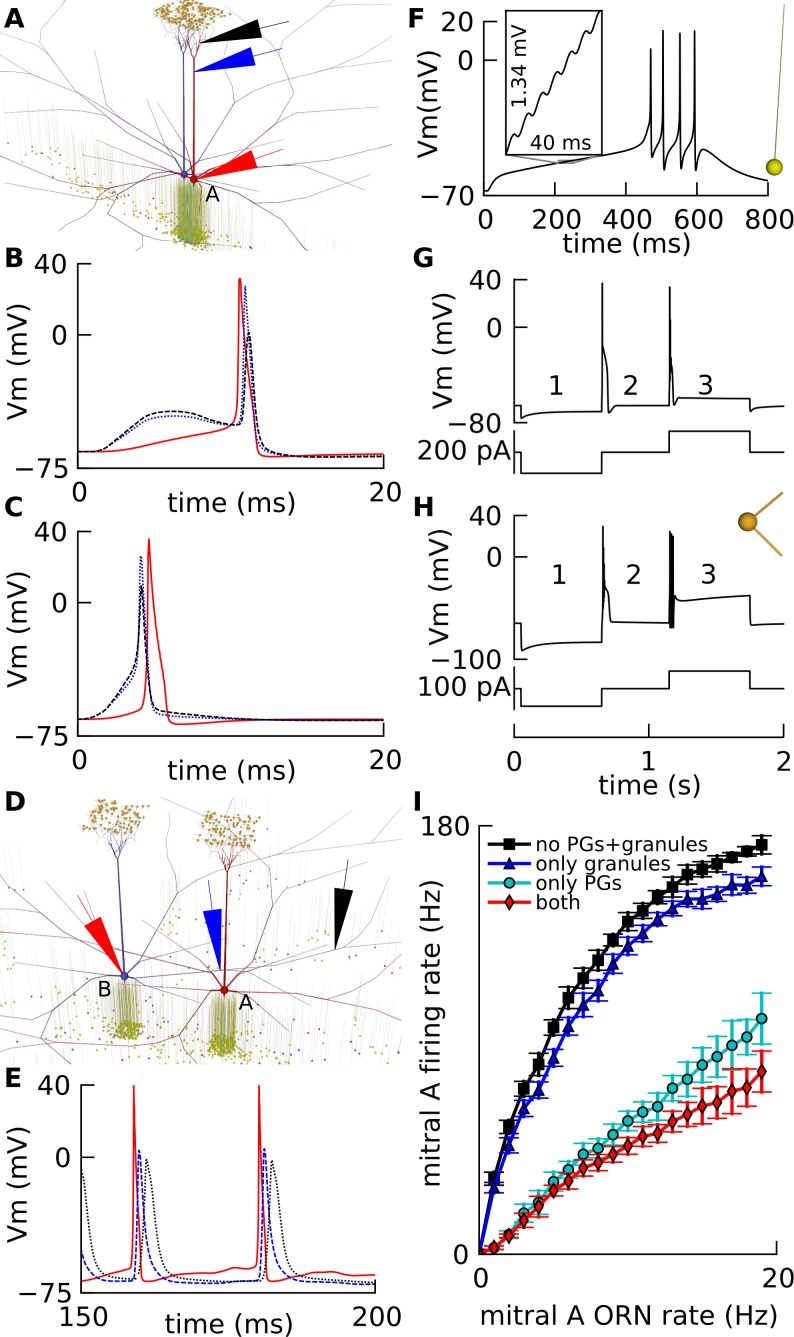
Single neuron model electrophysiological properties. Mitral cell: A. Visualization of a simulated *slice* network. We simulated ORN shock input to mitral cell A and its associated PG cells (Materials and Methods), while recording voltages in soma, base of tuft and tuft compartments (large arrows). B. Weak shock: action potential started at the soma (red solid) and spread to tuft base (blue dotted) and tuft (black dashed). C. Strong shock: action potential started at the tuft (black dashed) and propagated forward to tuft base (blue dotted) and soma (red solid). D. *Default* network *in vivo*, with mitral cells B and A, 400 μm apart (Materials and Methods). Recording electrodes are shown on mitral cell B at three locations. E. Spike propagation of cell B in circuit in D shown by voltage: at B’s soma (red solid), at the site of maximal inhibition on its lateral dendrite near soma of A (blue dashed), and farther along the same dendrite (black dotted). F. Granule cell physiology: Voltage at soma of granule cell (morphology at right) with a mitral → granule excitatory post-synaptic potential (EPSP) event delivered every 6 ms in a train totaling 100. This made the cell fire after a long latency. Inset shows integration of EPSPs. G, H: PG cell physiology: Somatic voltages of two PG cell models (same morphology at right) showing: (1) depolarizing ‘sag’ on hyperpolarization; (2) rebound burst with shoulder on recovery; and (3) low-threshold spike in G, or burst with plateau in H, on current injection. I. Input-output curve of mitral cell without lateral inhibition: Firing rate output of mitral cell A versus ORN firing rate input to its glomerulus, without input to mitral cell B, in *default* network shown in D with: all cells present (red diamonds); granule cells removed (cyan discs); PG cells removed (blue triangles); all interneurons removed (black squares).

We first modeled three cell types based on physiological data. Mitral cells were modeled with 286 compartments including 7 voltage-gated ion channels and calcium dynamics distributed over the cell, adapted from the Bhalla and Bower model [48]. Action potential could initiate at the soma for weak receptor input, and at the tuft for stronger input as observed [47] (Fig 2A–2C). Also, spikes could propagate along its lateral dendrite, despite localized inhibition [32] from granule cells receiving input from this and other mitral cells (Fig 2D and 2E).

Our two-compartment granule cell had a soma and a dendritic compartment, with Na, K and KA channels, adapted from Migliore and Shepherd’s model [49]. We set the channel densities to obtain a high spike threshold of ~25 mV above rest, as experimentally observed [50,39]. Thus the cell required a number of closely-spaced excitatory post-synaptic potentials (EPSPs) to fire, after a relatively long latency, as shown in Fig 2F.

Periglomerular (PG) cells had 3 compartments with 5 types of channels, and calcium dynamics. Two types of PG cell firing, namely low-threshold spiking and plateauing, have been reported [23]. We therefore constructed two PG cell models that qualitatively matched the two types of spiking (Fig 2G and 2H), by varying channel densities, but retaining the same morphology comprising a soma and two dendrites. The two PG cell models were incorporated in the ratio 67% low-threshold firing and 33% plateauing as observed [23].

Thus, each of the mitral, granule, and PG cell models were able to replicate basic electrophysiological properties.

### ORN, PG and mitral firing rates constrain glomerular synaptic strengths

Using these cell models, we constructed our network with physiological cell and synapse numbers, and set the synaptic strengths and time constants from evoked / spontaneous post synaptic potentials / currents reported in the literature (Table 1).We adjusted the glomerular ORN → mitral, ORN → PG and PG ←┤mitral connection strengths (Table 2) to match mean mitral cell firing rates for air and odor (1% saturated vapor) recorded extracellularly [18,16], given typical ORN firing rates [41,42], and examples of experimentally observed PG firing [44,45].

We simulated the input-output curve of a mitral cell *in vivo*, set within a *default* network, in the presence and absence of different inhibitory components (Fig 2I). This was consistent with the strong inhibitory effect of PG cells on mitral firing [46].

Thus, at this stage, we had parameterized the glomerular synaptic strengths and also the input-output relationship at two points for mitral cell firing (air and odor means).

### Inhibition between mitral cell pairs constrains the proximal mitral-granule synaptic strengths

**Fig 3:**
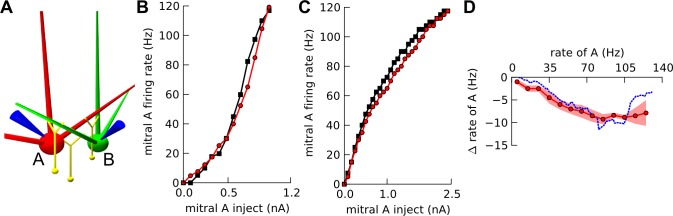
Short-range activity dependent inhibition between mitral cells. A. Schematic of model and experiment: Inhibition on mitral cell A due to mitral cell B ~50 μm apart is probed by simultaneous dual patch recordings [37]. B. Re-plotted experimental data [37] for a pair of mitral cells A and B *in vitro*. Firing output of A in response to current injection in A, in the absence (black squares) and presence (red discs) of simultaneous current injection in B (making B fire at ~80 Hz). C. An example simulation showing similar activity dependent inhibition as in B. The vertical separation between the curves is similar for B and C, but since the shape of mitral f-I curves can be very different [51], we did not match it for the example cell in B. D. Activity dependent inhibition showing mean change in firing rate of A due to fixed current injection in B, versus A’s firing rate. Blue: experimental data [37] re-plotted (mean over 15 inhibiting pairs out of 29 probed), Red: simulated mean change with SEM (over 5 most-inhibiting of 10 pairs generated by different network seeds).

We next parameterized the mitral-granule dendro-dendritic circuitry from observed lateral inhibition between nearby mitral cells, which is thought to be mediated by shared granule cells [37]. Arevian et al [37] patched simultaneously onto two mitral cells, say A and B, ~50 μm apart, in mouse olfactory bulb slices (Fig 3A). They found that evoked activity in cell B reduced the activity in cell A in 15 of 29 pairs (example in Fig 3B, mean in Fig 3D), but only when both A and B were firing at intermediate rates. Further, the onset of inhibition occurred sooner with greater summed mitral firing of A and B.

We replicated these experiments in a *slice* version of our default model (Table 3). We replicated the observed inhibition onset by adjusting the mitral → granule synaptic strength, since the spike latency of shared granule cells depended on total input rate (Fig 2F) from both mitral cells. We then set the granule ─┤mitral strength to replicate the observed mean amplitude of inhibition between A and B (example in Fig 3C, mean over multiple instances in Fig 3D).

**Table 3:** Summary of network connectivity patterns used in different variants of the olfactory bulb model.

Connectivity	Description	# of lateral glomeruli	Modifications	Inputs
***Random*; Figs 4A and 8A**	Mitral cells randomly rotated.	2	None.	ORN inputs scaled to get similar mean mitral firing as *default*. 35 Hz *in vivo* background to granule cells [39].
***Directed*; Figs 4B and 8B**	Central mitral sisters had dendrites from different lateral mitral cells pass near their somas.	2	(i) None. (ii) Leak reversal potentials of sisters at -58 mV and -70 mV.	As above
***Default*; Figs 4C and 8D**	B ‘super-inhibited’ A denotes: (a) B’s lateral dendrite passed near A’s soma; (b) 100 extra shared granule connections with A, proximal to B’s soma; (c) mitral → shared-granule strengthened 3× distally; (d) shared-granule ─┤ mitral strengthened 4×. Lateral mitral cells differentially ‘super-inhibited’ the two mitral cells of the central glomerulus. Most connections remained as in the *random* network (Discussion).	2 *(default)*; 6; or only 2 mitral cells A and B, with B super-inhibiting A (*default* for mitral input-output curve simulations).	(i) None. (ii) lateral mitral cells removed. (iii) PGs cells removed. (iv) granule cells removed. (v) all interneurons removed. (vi) non-linear input.	ORN inputs set to obtain mean mitral firing of ~10–15 Hz for odor and ~8 Hz for air. 35 Hz *in vivo* background to granule cells [39].
***Slice* / *in vitro*: based on *default* network; Figs 2A and 3A**	Two mitral cells A and B, 50 μm apart. Granule cells farther than 100 μm from the plane containing primary dendrites of A and B were discarded.	1	Lateral dendrites of A and B oriented randomly (most connections in *default* network are as in *random*).	Current injected into somas of A and B. *In vitro* background of 3.45 Hz to granule cells [52].

Most of the shared granule cells between nearby mitral cell pairs were proximal to both their somas, due to maximal overlap of the mitral cells’ dendrites in this region. Thus, this activity-dependent inhibition between nearby mitral cells [37] constrained the proximal mitral├→ granule synaptic strengths. The distal granule ─┤mitral synaptic strength was set from its observed distal decay [32] (‘Synaptic strengths’ sub-section in Materials and Methods), but the distal mitral → granule synaptic strength had to be parameterized by circuit-level constraints (decorrelation sub-section below).

Thus these pairwise recordings helped to define the crucial mitral├→ granule synaptic strengths for the model.

### Lateral dendrites deliver rather than receive inhibition

We next addressed a long-standing question in the field about the computational role of lateral dendrites of mitral cells: are they input or output structures? This possible dual role emerges from the observation that dendro-dendritic synapses with granule cells are bi-directional [7,53]. To address this question, we considered three key aspects of lateral dendritic inhibition between separated mitral cells A and B: (1) do lateral dendrites support back-propagating action potentials?; (2) how is mitral firing affected by the distance of granular inhibition along its lateral dendrite?; and (3), what kind of connectivity enables a mitral cell to strongly inhibit a distant mitral cell?

#### Spikes back-propagate along the lateral dendrite

In our *default* network, action potentials from the soma of mitral cell B, back-propagated along its lateral dendrite, even with inhibition from granule cells which were activated by background mitral firing and a strongly firing mitral cell A mid-way (Fig 2D and 2E). Thus, spikes propagated along the lateral dendrites of A and B, activating shared granule cells, which reciprocally inhibited A and B. This is consistent with experimental reports of attenuated [32,54,55] and unattenuated [56] back-propagation, despite focal inhibition [32]. We think that attenuation in propagating spike amplitude will not qualitatively modify our results, and can be folded into an increase in the mitral → granule strength with distance (see sub-section below: ‘Directed inhibition via a cluster of granule cells can mediate long-range inhibition’). With stronger activation of granule cells by multiple mitral cells, say at higher odor concentrations, gating of spikes may occur similar to experiment [56]. See also the sub-section in Materials and Methods: ‘Consistency of granule ─┤mitral decay with gating of spikes on lateral dendrites’.

#### Granule cell inhibition of mitral firing decreases with separation

**Fig 4:**
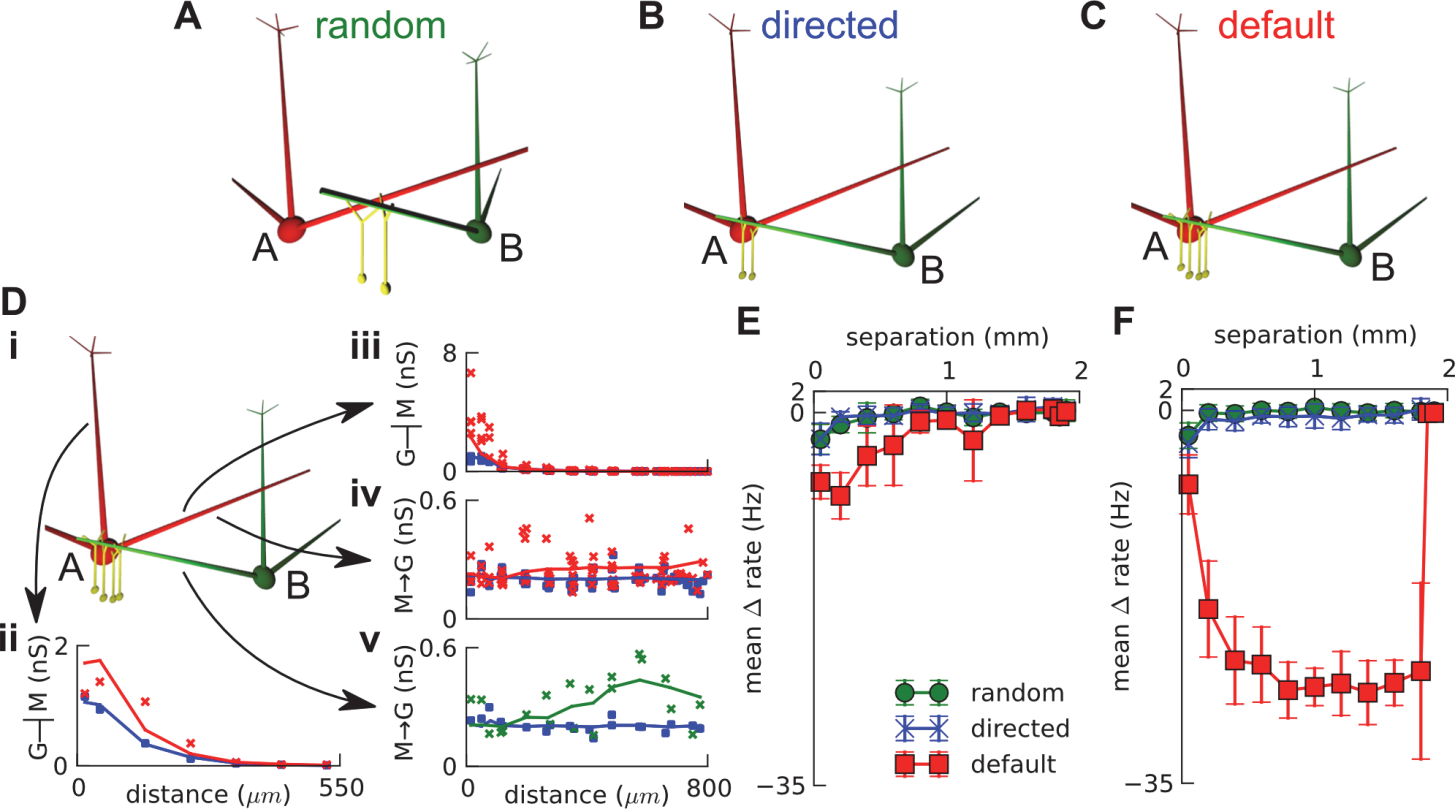
Long-range inhibition between mitral cells. A-C. Connectivity patterns: Central mitral cell A in red, and lateral mitral cell B in green, with a few shared granule cells (PG cells not shown). A. *Random* connectivity schematic: mitral cells’ dendrites are randomly rotated and B’s dendrites usually do not pass near the soma of A, leading to few and distal shared granule cells. B. *Directed* connectivity schematic: a dendrite of lateral mitral cell B is oriented to pass near soma of A, leading to a few shared granule cells proximal to A’s soma. C. *Default* / ‘Super-inhibitory’ connectivity schematic: Building on directed connectivity, extra shared granule cells with strengthened synapses are recruited between A and B, proximal to A’s soma. Synaptic strength distributions: D(i). Schematic of *default* network (same as C) where lateral mitral cell B ‘super-inhibits’ central mitral cell A. D(ii-v). Mean strength of synapses on a mitral cell compartment as a function of distance from soma. Blue lines and markers are for *random* and *directed* connectivity, while red/green are for *default* (i.e. ‘super-inhibitory’) connectivity. Solid lines represent means across 10 network seeds for a given connectivity; scatter plots are for a specific seed. (ii). Inhibitory granule —┤mitral synapses along the primary dendrite of mitral cell A. (iii). Inhibitory granule —┤mitral synapses along all lateral dendrites of mitral cell A. (iv). Excitatory mitral → granule synapses along all lateral dendrites of mitral cell A. (v). Excitatory mitral → granule synapses along the lateral dendrite of mitral B, which is super-inhibitory on mitral cell A. Long range activity dependent inhibition *in vivo*: Both mitral cells A and B receive ORN input as Poisson spikes on their tufts. E. Inhibition on B due to A: mean change in firing rate of B (mean across 0–19 Hz ORN inputs to B, and across 10 network instances—Materials and Methods), due to 10Hz ORN input to A, as the separation between cells A and B is increased for: *random* (green circles), *directed* (blue crosses), and *default* (red squares) connections. F. Inhibition on A due to B: as for E, but with A and B interchanged.

To address inhibition between mitral cells versus their separation, we examined three connectivity patterns: *random*, *directed*, and *default* (Fig 4A–4C and Table 3). The *random* network was built by randomly rotating the mitral cells around their primary dendrites, so that their lateral dendrites were oriented arbitrarily. Then 10,000 reciprocal synapses to granule cells were placed along the dendrites and soma of each mitral cell. For each reciprocal mitral├→ granule synapse on a mitral cell, its corresponding granule cell was chosen from a 100 μm × 100 μm area (granule dendritic extent) around the synaptic location on the mitral cell. Thus a few granule cells ended up being shared between any two mitral cells (Fig 4A).

The *directed* network differed from the *random* in that we oriented a dendrite of a lateral mitral cell to pass near the soma of a central sister mitral cell. Thus some shared granule cells ended up being close to the soma of the central sister (Fig 4B). The *directed* network differed from the *default* in that the number of shared granule cells and their synaptic strengths were at baseline for the directed connections.

The *default* network had oriented mitral dendrites similar to the *directed* network. Further, we connected extra shared granule cells near the soma of the central mitral cell. We increased the shared cells’ granule ─┤mitral synapses by 4 times, and the distal mitral → granule synapses from the directed dendrite onto shared granule cells, by 3 times. The spatial distribution of synaptic weights in these three networks is shown in Fig 4D. For details refer to Table 3 and subsection Materials and Methods: ‘Network construction and connectivity’.

We performed activity-dependent inhibition calculations between pairs of mitral cells, for each of these three networks. Since these were *in vivo* networks, we delivered a Poisson background of 35 Hz [39] onto granule cells to represent background network activity, and the stimulus was ORN input rather than current injection. We did these calculations for each network, for a range of separations of cell A and cell B, measuring mean effect of cell A on cell B (Fig 4E). In all cases the inhibitory effect on cell B decreased sharply with greater distance from the soma of B. Even in the *default* network having stronger mitral├→ granule connections onto a cluster of granule cells near A’s soma, the inhibitory effect on the soma of B remained weak.

There were three factors contributing to the reduction in inhibition on B due to A in all three networks: 1) drop in number of shared granule cells; 2) decay in the strength of the inhibitory granule —┤mitral connections away from the soma (Fig 4D(iii), Materials and Methods), as experimentally observed [32]; and 3) passive decay of somatic inhibitory post synaptic potentials as the ‘inhibition site’ went farther from B’s soma. Together these factors meant that inhibitory inputs onto lateral dendrites had little somatic effect except for synapses from the most proximal granule cells.

#### Directed inhibition via a cluster of granule cells can mediate long-range inhibition

We used the same three network configurations to examine how back-propagating action potentials along the lateral dendrite could mediate long-range inhibition. We repeated the calculations for activity-dependent inhibition at increasing cell separations, but this time from B to A (Fig 4(i) and 4F). We found that the inhibitory effect of lateral cell B on central cell A for a ‘directed, super-inhibitory’ connection in the *default* network, increased with larger separation (Fig 4F). In contrast, the inhibition fell in the *directed* and *random* networks. There were two factors leading to increasing inhibition on A due to B in the *default* network. First, as described above, B was less inhibited at greater separations, and this led to greater excitation of the shared granule cells proximal to A. This effect compensated for the reduction in number of shared granule cells with separation. Second, in the *default* network, the ‘super-inhibitory’ directed connection had 3× stronger synapses distal from B’s soma, to the shared granule cells near A’s soma (Fig 4D(v)).

Thus in our model, the mechanism for a lateral mitral cell B to strongly inhibit a distant mitral cell A, was by B’s action potentials propagating along its lateral dendrite and activating the column of granule cells on the soma, primary and proximal-secondary dendrites of A, where the granule —┤mitral synapses were strongest (S1 Video). Note that this connection was asymmetrical: B strongly inhibited A, but not vice-versa (Fig 4E and 4F).

It is interesting to note that our model brings together two experimental observations, namely the decay of granule ─┤mitral synapses [32], and the presence of reciprocal synapses on the primary dendrite [57] and soma [58], to infer columns of strongly-connected granule cells around the primary dendrites of mitral cells. This circuit prediction matches anatomical observations using retro-viral tracing [59], assuming that the virus travels preferentially across active or stronger synapses.

Thus, our model proposes that the distinctive, elongated mitral cell lateral dendrites deliver selective, long-range inhibition via back-propagating action potentials.

### Model replicates linear summation of odor responses

**Fig 5:**
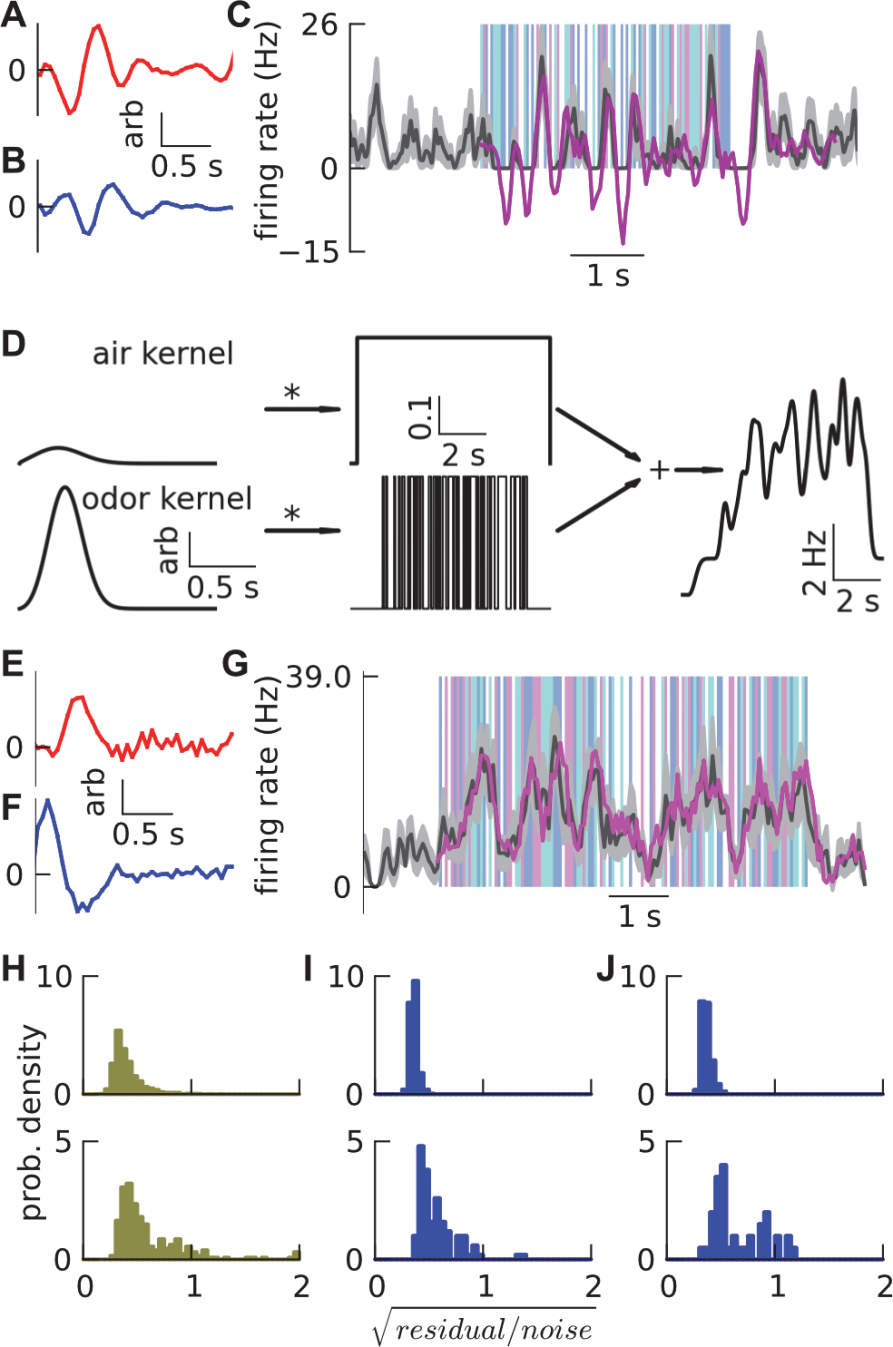
Linearity. A-C. Experimental example. (Re-plotted from data [17]): A-B. Two mitral odor kernels. Each kernel was obtained by linear fitting (least squared residual) of mitral responses to single-odor random pulse-trains (S2 Fig). C. Predicted response (magenta) using above kernels, to overlapping binary-odor pulse-trains (background bars in translucent red and blue), matches the mean mitral firing rate response (black line) shown with standard error of the mean (SEM) (gray width) over 12 trials. D-G. Model of linearity experiment. D. Model input. Air kernel and odor kernel (shared scale bars) were convolved with a constant suction pulse of air and an on-off random pulse-train of odor respectively, and added (along with a constant background) to generate the firing rate waveform of ORNs. The kernels have arbitrary units, as they are convolved with pulsed air-flow rate and odor-concentration pulse-trains, which have normalized units (to obtain mean air and odor firing rates), yielding ORN firing rate in Hz. E-G. Model results: Simulated example in *default* network, analogous to A-C above, except mean and SEM are over 9 trials. H-J. Goodness of linear fits and predictions: Fits / predictions with $\sqrt{residual/noise}\;<\;1$ are acceptable. Distribution of $\sqrt{residual/noise}$ for (top) fits of mitral responses to single-odor pulse-trains, and (bottom) predictions of responses to pulses of two odors overlapping in time. Last bin also contains all higher values. Standard deviation (SD) not SEM was used to calculate *noise*. H. Experimental data [17] re-plotted. I-J. Simulation results with: I. purely excitatory-component ORN kernels in 50 instances of *default* network (200 mitral-odor fits, 100 mitral-binary-odor predictions); and J. mixed i.e. excitatory- and inhibitory-component ORN kernels in 20 instances of the *default* network (80 mitral-odor fits, 40 mitral-binary-odor predictions). See also S2–S7 Figs.

Having parameterized the model, and replicated several circuit-level observations, we now investigated whether it replicated results of *in vivo* experiments on olfactory coding of odor pulses and mixtures [17,16]. Gupta and Bhalla reported mitral odor responses in tracheotomized, anesthetized rats, to random on-off pulses of odor delivered against a background of constant air-flow [17]. They found that responses of mitral cells to these odor pulses could be fit by a linear kernel (i.e. impulse response) convolved with the time-profile of the input odor’s concentration (example in S2 Fig). Further, they could predict the response to overlapping random pulse-trains of two different odors, as the rectified sum of the convolutions of each odor’s fitted kernel with that odor’s concentration-time-profile (example in Fig 5A–5C).

#### Simulated odor input

To simulate odor responses, we generated input ORN responses that were linear with concentration and flow rate. For the ORNs of each glomerulus, we first randomly generated different linear kernels (impulse responses) for air and two odors, as Gaussians in time (latency-to-peak = 150 to 350 ms, width = 250 to 450 ms). These were used to generate the random on-off pulse single-odor ORN input (Fig 5D) for our linearity simulations. This process was extended to generate overlapping pulses of two odors.

For completeness, we also tested: (1) a sigmoidal output non-linearity appended to the linear ORN model (S7H Fig); and (2) ORN kernels having a difference of Gaussians temporal profile mimicking excitatory and inhibitory components (Fig 5J).

#### The model predicts linear summation of mitral responses to pulsed odor input

Fifty random instances of the *default* network were simulated, with each glomerulus receiving pulsed odor input (peak pulse concentration = 1% saturated vapor) convolved with different random kernels as described above. An example simulation’s fits and prediction are depicted in S2 Fig and Fig 5E–5G respectively. Experimental (Fig 5H) and simulated (Fig 5I and 5J) distributions of $\sqrt{residual/noise}$ matched qualitatively both for single-odor response fits and binary-odor response predictions.

We also ran simulations with peak 2% saturated vapor. The fits and predictions were comparable to those from experiments with 1% saturated vapor, but the corresponding kernels at 1% differed in temporal structure from those at 2% (S3 Fig) similar to experiment [17].

**Fig 6:**
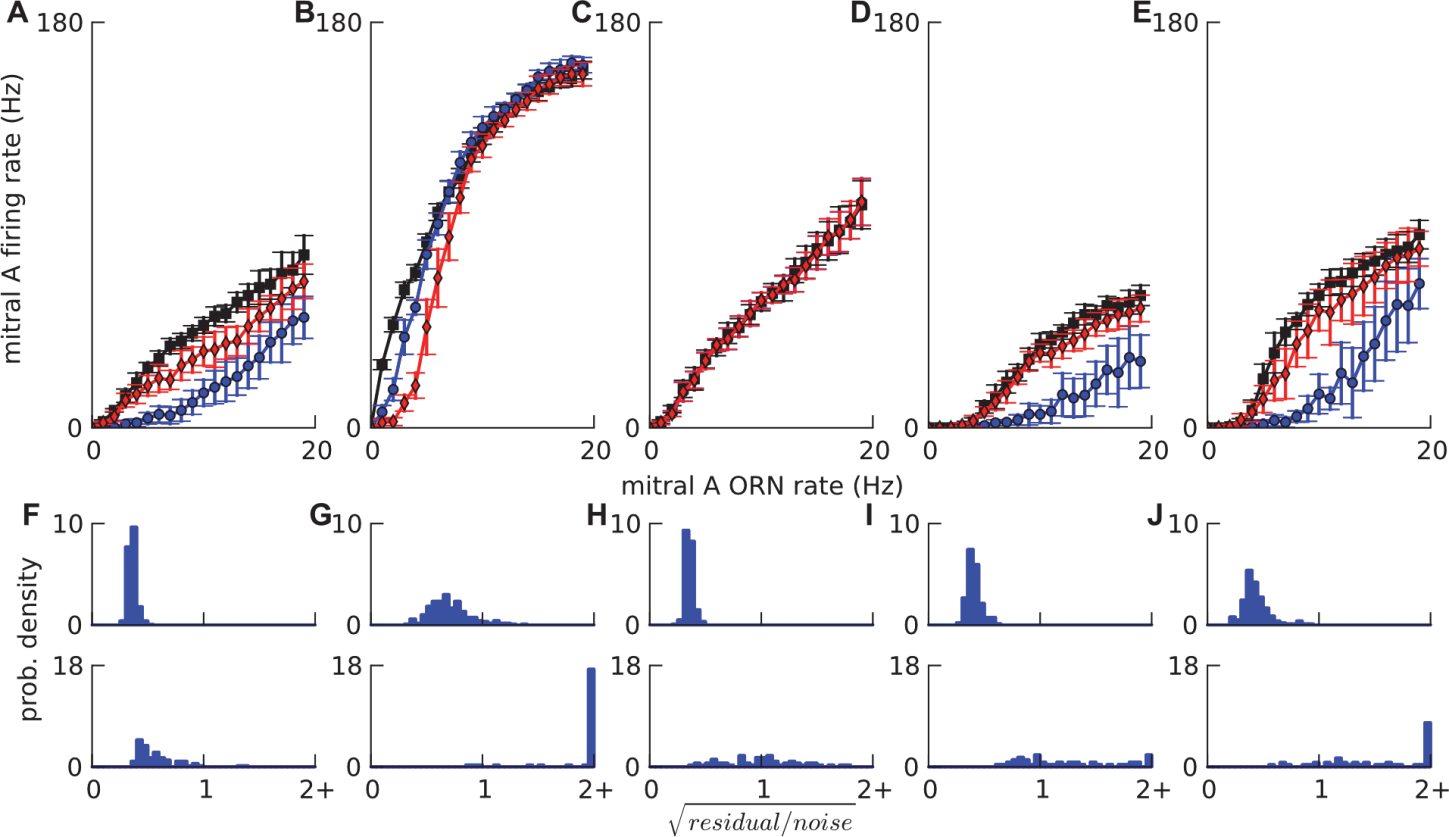
. Factors affecting linearity. A-E: Mitral input-output curves simulated in a *default* network (Figs 2D and 4D(i)) and modifications thereof. In each case mitral firing rate is plotted against ORN firing rate to the tuft of mitral cell A. Activity-dependent lateral inhibition to A, mediated by granule cells, is assessed in each case by considering a lateral ‘super-inhibiting’ mitral cell B receiving Poisson ORN input at 0 Hz (black squares), 5 Hz (red diamonds) and 10 Hz (blue circles). A. *default*. B. PG cells removed. C. granule cells removed. D, E: The *default* network is modified to have stronger PG cell excitation yielding half-Mexican-hat profiles i.e. suppression of mitral firing for low ORN input. D. ORN→PG and mitral→PG synaptic strengths are increased by 2.4 times for both plateau-ing and low threshold spiking PG cells. E. ORN→PG and mitral→PG synaptic strengths are increased by 6 times for plateau-ing PG cells but unchanged for low threshold spiking PG cells. F-J. The histograms of $\sqrt{residual/noise}$ for fits to single odor random pulse-trains (top row) and for predictions to two-odor random pulse-trains (bottom row) corresponding to above A-E cases respectively, from 30 network instances, each with different two odors. All values for $\sqrt{residual/noise}>2$ are in the right-most bin. The mitral input-output curve saturated to a small extent on removing granule cells (C) causing a worsening of the linear fits and predictions. But on removing PG cells (B), the saturation was much stronger and the lateral inhibition for 10 Hz input to lateral mitral cell B was less than that for 5 Hz. On modifying the input-output curve to have an initial supra-linear region (half-Mexican-hat) (D,E), the predictions were often seen to be supra-linear compared to the fits. Also, the lateral inhibition turned on with a threshold, i.e. negligible for 5 Hz input to mitral cell B but large for 10 Hz, since the lateral mitral cell B had the same non-linear input-output curve.

Here, the ORN input underwent an approximately linear transformation by the mitral cell’s ‘dynamic’ input-output function (averaged over 400 ms in Fig 2I) and was dynamically shaped by lateral inhibition from multiple mitral cells (averaged over 400 ms in Fig 6A) to yield the mitral response (and kernel). PG cells and granule cells fire earlier with stronger input, and thus dynamically change the temporal shape of the mitral kernel at higher concentrations. Overall, a mitral cell’s output was a limited-linear temporal combination of an excitatory primary input and a few inhibitory inputs from the glomerular representation.

#### The model predicts linear summation of mitral responses to respiration-sampled odor input

**Fig 7:**
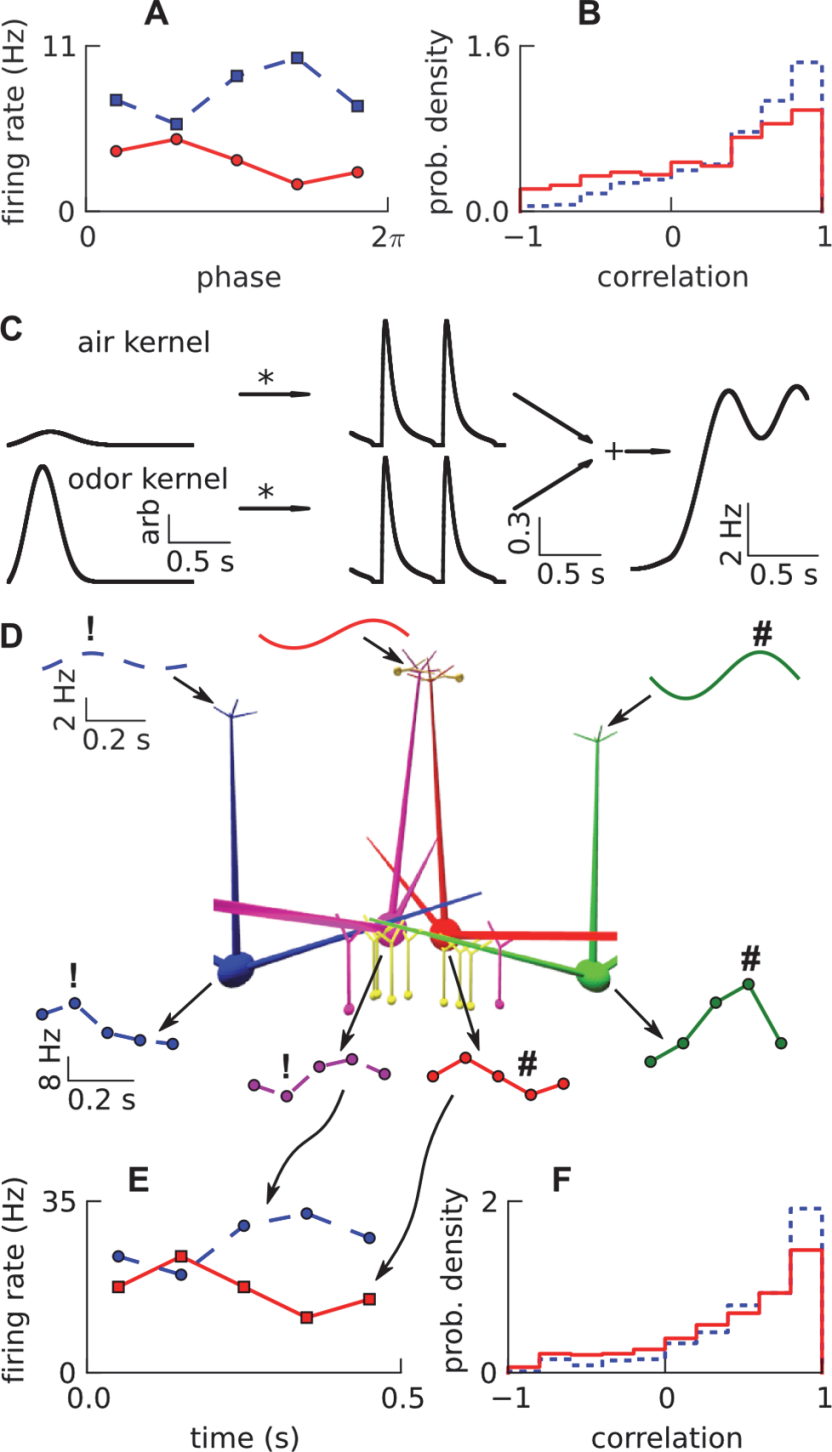
Phase decorrelation. A. Experimental odor responses of two sister mitral cells (squares vs discs), periodic with respiration, that were negatively correlated, re-plotted from data [18]. Firing rate is only approximate since we obtained respiration phase data instead of time data from [18]. B. Distribution of phase-correlations between responses of sister mitral cells to air (dotted) and odor (solid), (840 sister-pair—odor combinations, neglecting zero responses) re-plotted from data [18]. C. Respiratory odor input: air and odor kernels were convolved with ~2 cycles of rectified respiratory waveform, and added (along with constant background) yielding firing rate of ORNs in freely-breathing condition, for an odor input at a glomerulus. The kernels have arbitrary units, as they are convolved with air-flow rate and odor-concentration profile, which have normalized units (to obtain mean air and odor firing rates), yielding ORN firing rate in Hz. D. Proposed mechanism of decorrelation by super-inhibitory i.e. *default* connectivity with ORN firing rates to glomeruli for 1 respiration cycle (top), and subsequent mitral responses (bottom). The central mitral sisters received same excitatory input from ORNs, but differential inhibition from lateral mitral cells, at different phases of the respiration cycle (denoted by! vs #), which caused their outputs to be phase-decorrelated. E. Simulated responses of two sister mitral cells, in a *default* network instance, analogous to the experimental responses in A. F. Correlation distribution of simulated responses of sister mitral cells (350 sister-pair—odor combinations, neglecting zero responses) using same analysis as in B.

We also simulated freely breathing mitral responses using the same ORN kernels for 1% saturated vapor, convolved with a periodic respiration waveform as input (Fig 7C). Using mitral kernels obtained above by fitting random pulses, we were able to predict mitral freely breathing responses (S4 Fig), as in the experiment [17]. We also replicated another linearity experiment [16] in fitting the response to a mixture of two odors using only fitted responses for individual odors and air (S5 Fig).

Thus, with physiological settings of PG and granule cell inhibition, our model replicated two freely-breathing anesthetized rat experiments, demonstrating linear odor coding by mitral cells for respiration tuned input also.

#### Linearity is compromised over larger concentration range

We next asked if mitral responses were linear over larger variations in concentration. We simulated the responses to odor pulses of 200 ms duration at 1/3, 2/3, 1, 2 and 5% saturated vapor, generated similar to random pulses (Fig 5D).

The responses at higher concentration had a shorter latency to first spike after odor onset (S6A Fig), similar to experiment [39]. They also peaked earlier (earlier phase) due to faster rise time with inhibition kicking in stronger and earlier (with ORN input having only excitatory component) (S6B Fig).

Responses at concentrations different from 1% were decorrelated i.e. changed time profile, from the 1% response (similar to that seen with pulse trains above), and saturated at high concentrations (S7 Fig).

Thus, the network exhibited degraded linearity when probed with pulses of odor scaled over a wide concentration range, even though it remained linear when summing pulse-trains at any given fixed concentration.

### PG cells are the key circuit elements in shaping mitral response linearity

We next manipulated the inhibitory circuits in the *default* network to assess their contributions to the mitral cell input-output function, and the linearity and summation of pulse trains. For each manipulated network, we re-ran the *in vivo* activity dependent inhibition simulations and the random pulse-train simulations (1% saturated vapor) with linear kernel analysis. Removing PG cells drastically worsened the linear predictions because it induced non-linearity of both mitral input-output curve and lateral inhibition (Fig 6B–6G). Removing granule cells degraded linearity (Fig 6C–6H), but not as much as removing PG cells.

We tested stronger input to PG cells to create a half-Mexican-hat mitral input-output curve as proposed in some models [9,10]. (Fig 6D and 6E, 6I and 6J). These simulations suggest that PG cells play a more important role than granule cells in linearizing mitral input-output transformation.

Thus through the use of simulated cell knockout and circuit manipulation ‘experiments’, we found that PG cells are instrumental in linearizing olfactory bulb responses.

### Differential ‘super-inhibitory’ lateral connections phase-decorrelate responses of sister cells

Having analyzed emergent single-neuron coding, we next asked how cross-neuron coding features might emerge from the bulbar circuit. Specifically, Dhawale et al. found that ~30% of respiration-phase locked odor responses in sister mitral/tufted cells of freely breathing mice were negatively correlated [18], despite receiving similar excitatory input from their primary glomerulus (example in Fig 7A, correlation distribution in Fig 7B). Here, we first show that the ‘super-inhibitory’ lateral dendrite connectivity of our *default* model was sufficient to explain these features. In the next section, we show that this was the only circuit mechanism among several plausible ones that could fit the available data.

To simulate these experiments in the *default* network, we generated respiration-tuned ORN firing rates for each glomerulus by convolving odor and air kernels with a periodic air-flow respiration waveform as in Fig 7C. Sister mitral cells of the central glomerulus received similar input, while lateral mitral cells received different input, possibly peaking at different phases in the respiration cycle (Fig 7D top). The differential connectivity of these lateral mitral cells to the central sisters enabled them to inhibit the sisters at different phases in the respiration cycle. This super-inhibitory and differential inhibition led to phase-decorrelation (as opposed to spike-time decorrelation [51]) of responses between the sisters (Fig 7D bottom, Fig 7E) (S1 Video).

Through model parameter exploration, we determined that the 3× distal strengthening of mitral → granule connections was necessary for the lateral mitral cell to excite shared granule cells at typical low mitral firing rates, and the 4× strengthening of granule ─┤mitral connections was necessary to strongly modulate central mitral firing. Recall that the mean proximal weights were constrained by activity dependent inhibition [37] (sub-section above).

The distribution of phase-correlations between the simulated odor and air responses of the sister pairs in 350 instances of the *default* network, is shown in Fig 7F and is comparable to Fig 7B. Similar to experiment, odor responses were more phase-decorrelated than air responses, since air responses were weaker and had lower recruitment of lateral inhibition. Further, the delta-rate correlation, which measures if the change in mean firing rate for odor compared to air, is in the same direction between the sisters across all odors, was ~0.65, comparable to the ~0.68 from experiment [18].

Thus, super-inhibitory differential lateral connectivity is a sufficient network explanation for multiple aspects of decorrelation between sister mitral cells connected to the same glomerulus.

### Super-inhibitory and sparse lateral connectivity is uniquely able to account for phase-decorrelation

**Fig 8:**
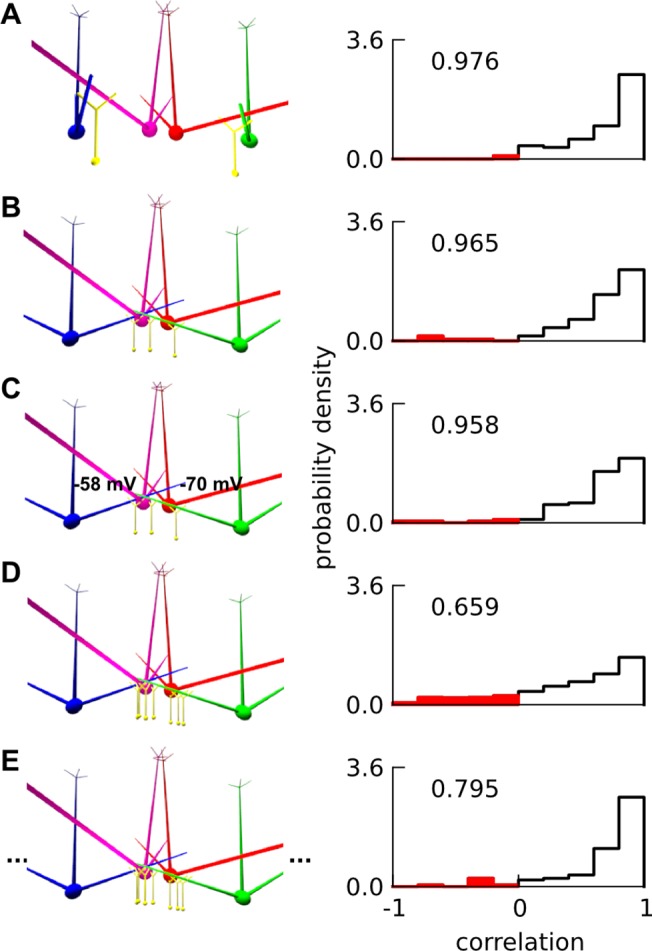
Mechanism of decorrelation. A-E. Distribution of correlations between phasic responses of two central mitral sisters (50 mitral-pair—odor combinations, but 350 for D) in different connectivities namely A. *Random* connectivity, B. *Directed* connectivity, C. *Directed* with different leak reversal potentials: -58 and -70 mV (effectively different thresholds) for the sister pair, D. *Default* ‘super-inhibitory’ connectivity with two lateral odor-responsive glomeruli, E. *Default ‘*super-inhibitory*’* connectivity with six lateral odor-responsive glomeruli. The delta-rate correlation is noted at top left of each histogram for each connectivity. For A-C, the receptor spike rate to central glomerulus was halved for odor compared to default i.e. D; while for E, it was quadrupled for air and doubled for odor, to get similar mean firing rate as D. Only the default model in D had substantial decorrelation: note the negative correlation bins, which have been shaded; and the delta-rate correlation values.

While we cannot rigorously show that our *default* (i.e. ‘super-inhibitory’) connectivity is *necessary* for phase decorrelation, we were able to rule out several alternative circuits. We simulated respiratory responses of central sister mitral cells in various connectivity schemes: namely *random*; *directed*; *directed* with leak reversal potentials of the central mitral sisters set at -58 and -70 mV; *default*; and *default* with 6 lateral glomeruli. Network schematics for these schemes and their simulated distributions of phase-correlations of sister odor responses are displayed in Fig 8. For each connectivity scheme we ensured that odor and air firing rates of central sisters corresponded to experiment. We maintained this by scaling the mean firing rate of the receptors, to compensate for changes in the inhibitory connections.

In all connectivities other than *default*, the means of the distributions of phase-correlations were much higher than experiment. Super-inhibitory connectivity with 2 lateral glomeruli yielded the correlation distribution closest to experiment (Fig 8D), while even super-inhibitory connectivity with 6 lateral glomeruli did not (Fig 8E). This is expected since different lateral glomeruli peak at different phases and thus their combined lateral inhibition would be spread more uniformly over the entire respiratory cycle. The experimental delta-rate correlation coefficient (previous section) was also matched only by the *default* connectivity with 2 lateral glomeruli. We have already shown above that lateral dendrites deliver rather than receive inhibition, thus the ‘super-inhibitory’ connections need to be proximal to sister mitral cells’ somas to phase-decorrelate responses.

In addition to granule-cell connectivities, we tested that intrinsic differences in resting potentials (hence different spike thresholds) between sister cells could not decorrelate responses (Fig 8C). While the scope of our model does not permit explicit exploration of the roles of short-axon or external tufted cells in decorrelation, our reading of the current literature suggests that they may not contribute (Materials and Methods).

Hence, from our simulations, we predict that there should be sparse, strong, proximal and differential lateral inhibition on sister mitral cells in order to decorrelate their responses to the extent observed experimentally.

## Discussion

We have developed a detailed compartmental model of olfactory bulb microcircuits incorporating a few odor-responsive glomerular column microcircuits and their interconnections. This model provides a mechanistic account of individual and cross neuron olfactory coding for identity, intensity, and mixtures. It specifically addresses the contrasting computational roles of mitral cell apical tufts versus lateral dendrites, especially their dendro-dendritic contacts, and supports the hypothesis that the lateral dendrites are primarily output structures. We have summarized the network-level constraints, replications of various experiments, and our predictions in Tables 2 and 1.

### Role of dendro-dendritic inhibition vis-a-vis other models

In a series of increasingly detailed models, Shepherd, Migliore and colleagues have looked at the generation of granule cell modules [60], their lateral inhibitory role in spike time synchronization and modulation [49,61,14,15] and in sculpting mitral spatio-temporal responses [15]. Cleland, Linster and colleagues have suggested that lateral inhibition is too weak to block spike generation but can modulate spike timing / synchronization [13,62,63], so much so that only intra-glomerular inhibition can perform olfactory decorrelation [10]. Davison, Feng and Brown also have models for spike time synchronization / modulation and related oscillations via granule cell inhibition [11,12].

We propose that, complementary to the above role of spike time modulation, granule cells mediate sparse and strong lateral inhibitory connections (currently unobserved) that can radically alter the respiratory phase of mitral firing compared to its primary glomerular input, causing phase decorrelation between sister mitral cells [18] rather than just spike-time modulation / decorrelation. Indeed, while spike-time decorrelation can be achieved by intrinsic cell variability [51], our simulations suggest that phase decorrelation cannot (Fig 8C).

In our study, we have only looked at phase decorrelation between sister mitral cells which receive the same excitatory glomerular input, not at phase decorrelation between mitral cells belonging to different glomeruli which in any case receive receptor input at different phases. While odor coding by spike latencies essentially between glomeruli has been studied in simplified models [64–66], the related phase coding (by phases of firing rate peaks) has not. It will be interesting to see the effect of non-redundant phase coding amongst sister mitral cells in a future model of spike latency or phase coding.

Based on our results, we expect phases of sister mitral cells to become highly correlated if granule cells are selectively silenced. If phase decorrelation is still seen, then other mechanisms must be considered, say (1) differential inhibition at the glomerulus, or (2) intrinsic differences between sister mitral cells. However, the former is unlikely given the averaging in the glomerulus via shared ET cells and gap junctions. For the latter, we specifically tested leak reversal potential (and hence spike threshold) as one intrinsic difference and did not find any resultant phase decorrelation (Fig 8C). Furthermore, intrinsic cellular differences are unlikely to lead to substantial differential phase shifts in the firing rate peak given similar averaged synaptic input.

In the spatial domain, the decrease in granule ─┤mitral strength with distance, coupled with the jump in mitral → granule strength beyond 100–200 μm, suggests that a major role of the lateral dendrites is to inhibit distal mitral cells. This distal inhibition is directed and sparse.

While granule cells play a primary role in phase decorrelation, PG cells linearize the mitral input-output curve. Hence if PG cells are selectively silenced, we expect strong saturation effects to show up in the mitral firing rate response versus odor concentration. Indeed, we expect PG cells to compensate for both receptor and mitral saturation / non-linearities. If linearity is maintained on silencing PG cells, then the ORN-mitral pathway may be fairly linear on its own, or the external-tufted—short-axon cell network may be involved in linearizing (see below). However, given the firing rates of PG cells, and their effectiveness in inhibiting the input at the tuft itself [46], we propose that silencing PG cells will uncover saturation and other non-linearities in the ORN and mitral responses.

Contrary to this, Cleland, Sethupathy, and Linster have proposed that intra-glomerular PG cell inhibition suppresses low mitral firing, causing a half-Mexican-hat mitral input-output curve, leading to contrast enhancement [9,10]. While we were able to replicate their proposal of half-Mexican-hat profiles with stronger PG excitation, this was at the cost of observed linearity (Fig 6D and 6E, 6I and 6J). We therefore think that the physiological role of PG cells is to achieve linearity rather than contrast enhancement.

We propose that the linearity of the feed-forward circuitry can be tested by light-activating axons of olfactory receptor neurons (which activate both mitral and PG cells) at a glomerulus at respiratory time-scales, and checking if the mitral rate response versus activation intensity is linear or not. The bulbar circuitry does offer alternative possibilities to obtain the observed linear summation [16,17]. For example, the short-axon cell network could dynamically linearize mitral responses. Dlx4/6 cells in zebrafish, the homolog of short-axon cells in mammals, depolarize mitral cells via gap-junctions for low-input and inhibit them (poly-)synaptically for high-input [67]. This could maintain the operating point on a sigmoidal input-output curve of the feed-forward micro-circuit close to its linear input-output regime for a given odor concentration, and thus dynamically linearize mitral cell odor responses as observed.

Thus our model proposes a testable role for the PG cells in linearization, and its falsification would implicate other mechanisms.

### Quantifying strong, sparse and differential inhibitory connections

We propose that lateral inhibition not only sculpts the mitral responses spatially and temporally, but also differentially between sister mitral cells, while maintaining limited linearity. Our prediction of strong, sparse and differential inhibition on sister mitral cells is corroborated by the recent observation of sparse lateral connections and the non-sharing of granule cells between neighboring or sister mitral cells (differential connections) [68,69]. We were unaware of these results at the time of model development; hence this is a strong convergence of findings. Further, our model predicts the strength, distribution (Fig 4D) and number (below) of these connections. This predicted connectivity also ties well with the theoretical result that strong, sparse connections lead to greater pattern decorrelation than weak, dense connections [70].

Based on our model and bulbar anatomy, we compute an upper bound and also a tighter, model-constrained estimate of the fraction of ‘super-inhibitory’ connections in the bulb. Given ~400 granule ←┤ M_1_ synapses proximal to a mitral M_1_’s soma in our model, and ~100 spines (reciprocal synapses) on a granule cell [27], we estimate 400×100 = 40,000 M_X_├→ granule ←┤M_1_ connections from any lateral mitral M_X_ to M_1_ proximally. For a mitral M_2_ to ‘super-inhibit’ M_1_, at least 100 proximal M_2_├→ granule ←┤M_1_ connections were required in our model. Thus, a maximum of 40000/100 = 400 lateral M/Ts can ‘super-inhibit’ M_1_, out of ~13000 M/T cells (~175 glomeruli × ~75 M/T cells per glomerulus) that can reach M_1_. We therefore predict an upper bound of ~1 in 33 M/T cells that can reach, and ‘super-inhibit’ M_1_.

From our model we found that super-inhibition on central mitral sisters from too few (0) or too many (6+) odor-activated mitral cells (of different lateral glomeruli) did not yield the observed decorrelation [18]. Hence we estimate that, for an odor at ~1% saturated vapor, on average only ~2 odor-activated lateral M/T cells effectively inhibit a given M/T cell M_1_ (consistent with experiment [71]), out of ~17 odor-activated glomeruli in dendritic range [4]. Thus a tighter estimate of the fraction of incoming ‘super-inhibitory’ connections on an M/T cell from others that can reach it, is ~2 in 17×75 i.e. ~1 in 640, smaller than the upper bound above. Since ~13000 M/T cells can reach M_1_, the mean fan-in i.e. number of incoming ‘super-inhibitory’ connections on an M/T cell is 13000/640 ~ = 20 (= fan-out).

These estimates of connectivity are strong quantitative predictions from our model that can be experimentally tested using retroviral and other tracing methods.

### Bulbar models and coding: beyond microcircuits

The experimental literature provides numerous constraints on bulbar anatomy and physiology at the cellular, micro-circuit and macro-circuit level. Our study synthesizes many such constraints, and marries these to odor response data from a range of *in vivo* experiments. We thus make experimentally driven and complementary predictions about coding mechanisms, to advance the scope of the evolving family of bulbar models [15,62,63,12].

We have been able to account for key, broad-brush coding features of mitral cells despite glossing over the role of several bulbar circuit elements. These circuit details may likely have network implications too. We did not model external-tufted, short-axon and other interneurons, nor gap junctions between mitral cells, and it will be interesting to see if future studies reveal a larger contribution to linearity or decorrelation from these components than our model suggests (Materials and Methods: ‘What was simplified in the model, and why’). We also did not include centrifugal modulation or learning, since our experimental references were in anesthetized preparations. We modeled only a few activated and connected columnar micro-circuits in the olfactory bulb, with the rest replaced by background activity to granule cells. Thus there is considerable scope to include more realism and detail; to study larger-scale functional organization, and to examine the roles of modulatory inputs, plasticity, homeostasis, and additional circuit elements.

Our model shows how many aspects of mitral coding emerge as the sum of a primary excitatory and a few lateral inhibitory temporal inputs from the glomerular representation. As an abstraction of our model circuit, the receptor neurons reduce the high-dimensional odor space to a lower-dimensional glomerular representation. Each mitral cell combines a few dimensions of the glomerular representation via coupled lateral mitral microcircuits. Sister mitral cells send different / decorrelated limited-linear combinations, disambiguating similar stimuli. Many mitral cells converge on each of the large number of pyramidal neurons in the olfactory cortex, mapping the glomerular representation somewhat linearly to the cortical one. Pyramidal neurons in the olfactory cortex require a number of possibly coincident (same phase) inputs from mitral cells to fire [72], thus thresholding the limited-linear transformation, enabling odor classification. Also, the reduction to and expansion from a low-dimensional bottleneck glomerular representation possibly enables feature extraction and other computations [73–75].

## Materials and Methods

### Simulator

We used the Multi-scale Object Oriented Simulation Environment (MOOSE, http://moose.ncbs.res.in/, moose_Beta_1.4 branch, svn commit 3207) with Python scripting [76] for all our simulations. We wrote the specifications of the network connectivity, cellular morphology and the kinetics of a few channels and synapses for our model in NeuroML 1.8.0 (neuroml.org) with minor custom extensions for spike train input and rotation of cells. The kinetics for the remaining channels and synapses were written as Python scripts (python.org) for backward compatibility reasons. We also wrote Python scripts to generate: (1) NeuroML files for multiple instances of network models starting from different random seeds; and (2) custom input files for different ORN kernels and Poisson spike trains for ORN and baseline granule input.

We extended MOOSE to load NeuroML files along with input spike trains. We farmed multiple trials with the same network instance, but different ORN and baseline granule cell input on a cluster. Simulation setup and post-simulation analysis code was in Python (python.org, scipy.org, mpi4py.scipy.org). Our model and related scripts can be downloaded from Senselab ModelDB (http://senselab.med.yale.edu/ModelDB/ accession number: 153574) and Open Source Brain (http://www.opensourcebrain.org/projects/olfactory-bulb).

### Biophysical model of microcircuits in the rat olfactory bulb

We constructed a biophysical model of coupled odor-responsive microcircuits in the rat olfactory bulb, using compartmental models for mitral, granule and periglomerular (PG) cells. Input from olfactory receptor neurons (ORNs) was represented as time-varying Poisson spike trains. These inputs were afferent onto mitral tufts and PG dendrites. Since we were interested in simulating odor responses of two sister mitral cells, we modeled a central glomerulus containing the two mitral cells of interest, and zero to six lateral glomeruli in its dendritic field, that were activated by odor and inhibited the central mitral cells.

At concentrations of interest (~1% saturated vapor), an odor activates glomeruli sparsely [4,71]. On average, a single odor activated 17 glomeruli in a region approximately 15 glomeruli in diameter (i.e. 17 out of ~177 glomeruli) in the rat [4]. Of the few number of glomeruli activated by an odor in the dendritic field of the two sister mitral cells, even fewer will be connected to the sisters (via shared granule cells). Thus we simulated up to a maximum of six odor-responsive lateral glomeruli, each having 2 mitral cells and corresponding PG and granule cells. In *default* networks, one mitral cell of each lateral glomerulus was connected strongly (‘super-inhibition’) and differentially, to one of the central sisters via shared granule cells.

In our model, PG cells mediated feed-forward (ORN → PG ─┤mitral) and recurrent (mitral├→ PG) intra-glomerular inhibition, and granule cells mediated self / recurrent (mitral├→ granule) and lateral (mitral1 → granule ─┤mitral2) inhibition. We used spatial scales and connectivity from rat, but we constrained the model and reproduced results from experiments on both rats and mice. We ignored centrifugal inputs to the bulb, restricting the model to experiments on anaesthetized animals, where centrifugal modulation is low [77,78].

A summary of cell numbers, synaptic numbers, modeling strategies for each, and experimental and simulated synaptic strengths and time scales is provided in Table 1.

#### Cell models

We used a modified version of Bhalla and Bower’s 286 compartment model of the mitral cell [48], using NeuroML morphology exported from its NEURON (http://www.neuron.yale.edu/neuron/) translation by [11]. Notably, the tuft and the primary dendrite were made more excitable, and the membrane time constant roughly halved to 50 ms by halving the membrane resistance. We added a special Na channel [49] in the initial segment to obtain spike initiation in the soma for weak input and in the tuft for strong input, inspired by another model [49].

Our two-compartment granule cell had a soma and a dendritic compartment, with Na, K and KA channels, adapted from Migliore and Shepherd’s model [49]. Most granule cells do not spontaneously fire action potentials *in vivo*, despite a high 35Hz barrage of EPSPs [39]. Their thresholds are known to be quite high: ~16 mV above rest (n = 6) [39] and ~25 mV above rest [50]. In addition granule cells are known to spike with a long latency ~350 ms [79]. Hence, the Na, K and KA densities were varied to set a spike threshold of ~25 mV above rest, requiring a number of closely-spaced EPSPs to make the cell fire after a latency of integration (Fig 2F). This integration was required to obtain the activity dependent inhibition observed *in vitro* [37].

PG cell models had a soma and two dendritic shaft compartments from the soma. Their resting V_m_ was set to −65 mV [24]. Two types of PG cells, namely plateauing and low threshold spiking, were constructed by adjusting the Ih, T-type Ca, K, and KA channels, to match properties seen in [23], inspired by a preliminary report [80], but independently of recent PG cell models [81,82] We matched three effects (Fig 2G and 2H) from [23]: (1) depolarizing ‘sag’ (due to Ih) on hyperpolarization with -100 pA injection in Fig 2G / -50 pA in Fig 2H; (2) rebound burst (TCa, Na) with delay (KA) and shoulder (TCa) on recovery to zero current injection; and (3) low-threshold spike (TCa) on current injection of 100 pA in Fig 2G, versus burst with plateau on current injection of 50 pA in Fig 2H.We used 33% plateauing and 67% low-threshold spiking PG cells [23].

We provide a summary of / references for the channel parameters in Table 4.

**Table 4:** Channel kinetics and parameters (temperature T = 35°C, relevant units for numerical values are specified).

Channel / Ion-Pool name (cell name)	Kinetics
**Na_mit_usb (mitral), K2_mit_usb (mitral, PG, granule), K_mit_usb (mitral), LCa3_mit_usb (mitral), KA_bsg_yka (mitral, PG), Kca_mit_usb (mitral), Ca_mit_conc (mitral, PG)**	Same as in Bhalla and Bower’s model [48]. For the granule and PG cells, the reversal potential for all K channels was set to -80 mV.
**Na_mit_initialsegment_MS (mitral): Same as Na channel in the initial segment of the mitral cell of Migliore and Shepherd** [49] **(translated from NEURON model available online as Senselab ModelDB accession number: 97263).**	*I*(*V*) = *m* ^3^ *h g*_*max*_ (50 *mV*–*V*), $\frac{dm}{dt}=\frac{m_{\infty }}{\tau_{m}}-\frac{m}{\tau_{m}},\;\frac{dh}{dt}=\frac{h_{\infty }}{\tau_{h}}-\frac{h}{\tau_{h}}$, $\tau_{m}\left(V\right)=\max\left(\frac{1}{\alpha_{m}\left(V\right)+\beta_{m}\left(V\right)},0.02\;ms\right),m_{\infty }\left(V\right)=\frac{\alpha_{m}\left(V\right)}{\alpha_{m}\left(V\right)+\beta_{m}\left(V\right)}$, $\tau_{h}\left(V\right)=\max\left(\frac{1}{\alpha_{h}\left(V\right)+\beta_{h}\left(V\right)},0.5\;ms\right),h_{\infty }\left(V\right)=\frac{1}{1+\exp\left(\frac{V+50\;mV}{4\;mV}\right)}$, *α*_*m*_(*V*) = *trap*(*V*,−30*mV*,4×10^5^ *s* ^−1^ *V* ^−1^,7.2*mV*), *β*_*m*_(*V*) = *trap*(−*V*,30*mV*,1.24×10^5^ *s* ^−1^ *V* ^−1^,7.2*mV*), *α*_*h*_(*V*) = *trap*(*V*,−45*mV*,3×10^4^ *s* ^−1^ *V* ^−1^,1.5*mV*), *β*_*h*_(*V*) = *trap*(*−V*,45*mV*,1×10^4^ *s* ^−1^ *V* ^−1^,1.5*mV*),$trap(V,th,a,q)=\{\begin{array}{c} a\frac{V-th}{1-\exp\left(-\left(V-th\right)/q\right)}2^{\left(T-24{}^{\circ}C\right)/10{}^{\circ}C}\text{ if }\left|V-th\right|>1nA\\ a\;q\;2^{\left(T-24{}^{\circ}C\right)/10{}^{\circ}C}\text{ if }\left|V-th\right|\leq 1nA \end{array}$
**Na_rat_ms (granule, PG): Same as Na channel in granule cell of Migliore and Shepherd** [49] **(translated from the NEURON model available online as Senselab ModelDB accession number: 97263).**	As above, except: *I*(*V*) = *m* ^3^ *h g*_*max*_ (60 *mV*–*V*), $h_{\infty }\left(V\right)=\frac{1}{1+\exp\left(\frac{V+35\;mV}{4\;mV}\right)}$, *α*_*m*_(*V*) = *trap*(*V*,−15*mV*,4×10^5^ *s* ^−1^ *V* ^−1^,7.2*mV*), *β*_*m*_(*V*) = *trap*(−*V*,15*mV*,1.24×10^5^ *s* ^−1^ *V* ^−1^,7.2*mV*), *α*_*h*_(*V*) = *trap*(*V*,−30*mV*,3×10^4^ *s* ^−1^ *V* ^−1^,1.5*mV*), *β*_*h*_(*V*) = *trap*(−*V*,30*mV*,1×10^4^ *s* ^−1^ *V* ^−1^,1.5*mV*)
**KA_ms (granule): From Migliore and Shepherd’s model** [49] **(translated from the NEURON model available online at Senselab ModelDB accession number: 97263).**	*I*(*V*) = *mhg*_*max*_(−90*mV*–*V*), $\frac{dm}{dt}=\frac{m_{\infty }}{\tau_{m}}-\frac{m}{\tau_{m}},\;\frac{dh}{dt}=\frac{h_{\infty }}{\tau_{h}}-\frac{h}{\tau_{h}}$, $\tau_{m}\left(V\right)=\frac{\exp\left(0.75\left(V+45\;mV\right)/10mV\right)}{3^{\left(T-24{}^{\circ}C\right)/10{}^{\circ}C}\;0.04\;ms^{-1}\;\left(1+\exp\left(\left(V+45\;mV\right)/10\;mV\right)\right)}$, $m_{\infty }\left(V\right)=\frac{1}{1+\exp\left(-\left(V-17.5\;mV\right)/14mV\right)}$, $\tau_{h}\left(V\right)=\frac{\exp\left(0.99\left(V+70\;mV\right)/5mV\right)}{3^{\left(T-24{}^{\circ}C\right)/10{}^{\circ}C}\;0.018\;ms^{-1}\;\left(1+\exp\left(\left(V+70\;mV\right)/5\;mV\right)\right)}$, $h_{\infty }\left(V\right)=\frac{1}{1+\exp\left(\left(V+41.3\;mV\right)/6mV\right)}$
**TCa_d (PG): From a thalamic reticular neuron model** [83,84] **(translated from the NEURON model available online at Senselab ModelDB accession number: 17663).**	*I*(*V*) = *m* ^2^ *hg*_*max*_(120*mV*–*V*), $\frac{dm}{dt}=\frac{m_{\infty }}{\tau_{m}}-\frac{m}{\tau_{m}},\;\frac{dh}{dt}=\frac{h_{\infty }}{\tau_{h}}-\frac{h}{\tau_{h}}$, $\begin{array}{r} \tau_{m}\left(V\right)=\left(3\;ms+\frac{1\;ms}{\exp\left(\left(V+25\;mV\right)/10mV\right)+\exp\left(-\left(V+100\;mV\right)/15mV\right)}\right)\\ \times \frac{1}{5^{\left(T-24{}^{\circ}C\right)/10{}^{\circ}C}} \end{array}$, $m_{\infty }\left(V\right)=\frac{1}{1+\exp\left(-\left(V+50\;mV\right)/7.4mV\right)}$, $\begin{array}{r} \tau_{h}\left(V\right)=\left(85\;ms+\frac{1\;ms}{\exp\left(\left(V+46\;mV\right)/4mV\right)+\exp\left(-\left(V+405\;mV\right)/50mV\right)}\right)\\ \times \frac{1}{3^{\left(T-24{}^{\circ}C\right)/10{}^{\circ}C}} \end{array}$, $h_{\infty }\left(V\right)=\frac{1}{1+\exp\left(\left(V+78\;mV\right)/5mV\right)}$
**Ih_cb (PG): From an experimentally-determined channel model** [85] **(translated from NEURON model available online at Senselab ModelDB accession number: 3665).**	*I*(*V*) = *l g*_*max*_(−30 *mV*–*V*), $\frac{dl}{dt}=\frac{l_{\infty }}{\tau_{m}}-\frac{l}{\tau_{m}}$, $\tau_{l}=\frac{\exp\left(\left(V+65\;mV\right)/23.529411765\;mV\right)}{0.85\left(1+\exp\left(\left(V+65\;mV\right)/11.764705882mV\right)\right)}\frac{1}{4.5^{\left(T-30{}^{\circ}C\right)/10{}^{\circ}C}}$, $l_{\infty }=\frac{1}{\left(1+\exp\left(\left(V+80\;mV\right)/10mV\right)\right)}$
**Kca_mit_usb_pg (PG)**	Same as Kca_mit_usb of Bhalla and Bower’s model [48], except that Ca^2+^ half-point in the Ca dependence factor was changed to 0.0055 mM instead of 0.015 mM.

#### Network construction and connectivity

For a given network connectivity (Table 3), we created multiple network instances using different random seeds, corresponding to coupled micro-circuits in the olfactory bulbs of distinct rats or distinct bulbar areas of the same rat. Since our model was limited to explaining single or coupled neuron responses, we modeled a central odor-responsive glomerulus with only two sister mitral cells, and 0 to 6 odor-responsive lateral glomeruli that may strongly influence the two central sister mitral cells of interest.

Hence, for a network instance, we first generated positions for a ‘central’ glomerulus and 0–6 ‘lateral’ glomeruli in an 850×850 μm^2^ area around it i.e. within mitral dendritic reach. Then we placed 2 mitral cells with their primary tufts in each glomerulus, and their somas in the mitral cell layer below. Due to computational constraints, we retained only two mitral cells per glomerulus. We rotated one mitral cell from each lateral glomerulus, so that one of its lateral dendrites passed close to alternately one or the other central sister’s soma (Figs 8B–8D, 4B and 4C), for the *directed*, *default* and *slice* networks but not for the *random* network (Figs 8A and 4A).

At each glomerulus, we placed 1000 PG cells in a 2D array. For each mitral cell, we created 100 reciprocal mitral├→ PG synapses, each between a randomly-chosen tuft compartment of the mitral cell in that glomerulus, and any of the two dendrites of a randomly chosen PG cell. We repeated these mitral → PG synapses, to complete 25 synapses per PG cell, distributing their delays uniformly from 0 ms to 40 ms. PG ─┤mitral synaptic delays were distributed exponentially with a mean of 160 ms.

We next created a granule cell layer with realistic density: 2500 granule cells per (100 μm)^2^, beneath the mitral cell layer. For each mitral cell, we formed 10^4^ reciprocal (mitral├→ granule) synapses [31,86] uniformly along the length of its primary [57] and secondary dendrites, with 80 on its soma [58]. For each reciprocal synapse on the mitral cell, the corresponding granule cell was chosen randomly from among those having somas within 100 fm × 100 μm (granule dendritic extent) of the synapse location on the mitral dendrite. Thus granule cells would get connected to 0, 1 or more of the modeled mitral cells.

Further, for the *default* network and its derivative *slice* network, we increased the inhibition between directed mitral cells. Thus for every ‘super-inhibitory’ pair of central sister and lateral mitral above, we randomly chose 100 granules cells connected only to the central mitral cell and on its primary dendrite, soma or proximal secondary dendrites. We then connected these granule cells to the closest segment of the directed dendrite of the lateral mitral cell (Figs 8D and 4C). Also, for every such pair, we strengthened all granule ─┤mitral synapses by 4 times, and all mitral → granule synapses which were more distal than ~100 μm by 3 times.

The strong proximal granule ─┤mitral synapses around each central sister’s soma due to super-inhibitory connections effectively created a ‘column’ of granule cells. We also strengthened the proximal granule ─┤mitral synapses of the lateral mitral cells to create columns of granule cells around them in lieu of additional lateral mitral cells ‘super-inhibiting’ these lateral mitral cells,

We modeled only those odor-responsive mitral cells that strongly inhibited the central sister mitral cells, via shared granule cells. We incorporated the average inhibitory effects of the large number of mitral cells that we did not model, by a 3.45 Hz *in vitro* [52] and a respiratory-tuned 35 Hz *in vivo* [39] Poisson background input to all modeled granule cells via a mitral → granule synapse. We were justified in this as the simulated *in vivo* activity dependent inhibition between randomly oriented mitral cells, in a *random* network, was negligible (Fig 4E and 4F).

We estimated synaptic numbers from reported numbers of PG & granule cells (vis-à-vis mitral cells), and their spine counts, assuming one connection per spine (Table 1). For example, 50 spines per PG cell × 1000 PG cells per glomerulus were connected to 50 M/T and 200 ET cells per glomerulus. Thus, each mitral cell was connected to 100 PG cells.

Leak reversal potentials of granule cells were spread normally with a standard deviation (SD) of 2.25 mV similar to experiment [39], truncated at 6 SDs on either side; while those of PG cells were spread normally with an SD of 3 mV similar to experiment [22], truncated at 8 SDs on either side. These spreads and spreads in synaptic strengths (sub-section below) contributed to asynchronous activation of interneurons.

The *slice* network was created from a *default* network having a central and a single lateral glomerulus. We pruned granule cells more than 100 μm away on either side of the plane containing the primary dendrites of the two mitral cells A and B (‘Inhibition between mitral cell pairs constrains the proximal mitral-granule synaptic strengths’ sub-section of Results). To replicate the close-range *in vitro* activity dependent inhibition [37], the soma of a mitral cell ‘B’ from the lateral glomerulus, was placed 50 μm away from a central sister ‘A’. Since most paired connections even in the *default* network were random, the dendrites of A and B were also randomly rotated, but there were granule columns around A and B as in a *default* network.

For the *in vivo* activity dependent inhibition, we used the *default* network but with only two mitral cells A and B. We varied the separation between A and B, and here B ‘super-inhibited’ A.

The different connectivities we probed are listed in Table 3 and discussed in Results. Numbers of cells and connectivities with sources are listed in Table 1.

#### Aggregation of non-shared granule cells

After creating the reciprocal mitral├→ granule synapses as above, unconnected granule cells were pruned. Shared granule cells connected to two or more mitral cells were left 1:1, and not aggregated, so as not to average out lateral inhibition effects. In the *default* network, we had ~1200 jointly or multiply connected granule cells between 6 mitral cells. But singly-connected or unshared granule cells, connected to only one mitral cell were aggregated 100:1, i.e. a hundred of them were replaced by a single granule cell. Even after 100:1 aggregation, the number of ‘singles’ were ~95 per mitral cell.

For each such 100:1 aggregated granule cell, the excitatory mitral → granule input synapse was maintained the same, but the inhibition to the connected mitral cell was increased corresponding to the effect of 100 granule cells. However, just multiplying granule ─┤mitral synaptic strength, would make this proxy inhibition too large and synchronous. So instead of a single synapse of 100× weight, we set up 10 synapses, each of strength 10×, with staggered delays, triggered by the same pre-synaptic granule cell spike. The staggered delays were distributed exponentially with a standard deviation of 160 ms similar to experiment [36,37].

We confirmed that simulations with 100:1 aggregation gave qualitatively similar results to 20:1 aggregation for activity dependent inhibition *in vivo* (with ORN input). Thus these aggregation ratios seem justified.

Typical model-construction approaches start out with a reduced number of cells and connect them up via proportionally stronger synapses. By contrast, we first created a model with realistic numbers of mitral, granule and PG cells, connected them up with realistic numbers of synapses, and then discarded, neglected, or aggregated cells based on their connectivity. The advantage with our method of cell number reduction is that (1) differential connectivity effects are not averaged out as only unshared granule cells are aggregated, not shared ones, (2) synaptic strengths and integration effects are partly maintained using non-synchronous scaling of synapses, and (3) proxy background (in our case excitation to granules cells) is provided in lieu of the discarded (mitral) cells.

#### Synaptic strengths

We modeled synapses as dual exponential conductances. Excitatory glutamatergic mitral→granule synapses had both AMPA and NMDA components. Magnesium-block voltage-dependence and NMDA to AMPA ratio were taken from experiment [33]. Inhibitory GABA-based granule ─┤mitral and PG ─┤mitral synapses had a single component as did excitatory ORN → mitral, ORN → PG and mitral → PG synapses. We set synaptic strengths and time constants from spontaneous / evoked EPSPs / IPSPs in whole-cell recordings, where available. However, synaptic strengths were modified from above putative settings to better obtain network effects like activity dependent inhibition, linearity and decorrelation as summarized in Table 2. A summary of synaptic numbers and weights is provided in Table 1.

All synapses in our model were spike-based. Dendro-dendritic synapses are usually considered graded, however the mitral → granule synapse was modeled as spike-based because in the presence of TTX, “graded depolarization [of the mitral cell] below threshold for calcium spike initiation failed to activate the reciprocal synapse” [87,88]. The granule ─┤mitral synapse has multiple modes of activation due to: (a) local Ca transients in the spine, (b) local Ca transients in the dendrite, or (c) global Ca transients due to spikes [50], which together possibly produce the graded effect.

We implemented the inhibitory effect of the Ca transient in the granule cell spine, on the mitral cell, by creating a weak auto-inhibitory synapse on the mitral cell, at each reciprocal mitral├→ granule synapse, which was activated when the mitral cell fired. The strength of this auto-inhibitory synapse should be the same as the inhibitory part of the reciprocal synapse as it is indeed the same synapse, just its activation is different. However, spinal neuro-transmitter release is expected to be stochastic and dependent on the number of pre-synaptic action potentials, whereas with our auto-inhibitory synapse all 10^4^ synapses per mitral cell get activated on each spike. Thus a weak, effective synaptic strength of 5 pS was used to avoid strongly inhibiting the mitral cell.

Apart from this, the usual granule ─┤mitral synapse in our model was spike-based, to represent the activation due to global Ca transient following a granule cell spike. We reduced granule ─┤mitral synaptic strengths exponentially along the dendrites (length constant 100 μm on primary and 150 μm on secondary dendrites), and further reduced them proportionally with diameter, both as measured experimentally (after space clamp correction) [32], and shown in Fig 4D(ii-iii).

For *default* connectivity (schematics in Fig 4C and 4D(i)), the reciprocal synapses mediating inhibition between the ‘super-inhibitory’ mitral cell pairs were strengthened: granule ─┤mitral synapses were made 4× stronger (Fig 4D(ii-iii)), and mitral → granule synapses were made 3× stronger distally compared to proximally from the mitral soma (Fig 4D(iv-v)). Mitral → granule synapses proximal to the mitral somas were not strengthened; else the self- and neighbor-inhibition became too strong and pegged firing at too low values. In any case, the proximal strength was constrained by activity dependent inhibition. The increased distal mitral → granule synapses were necessary to perform lateral inhibition to the extent needed for replication of decorrelation (Results). The mitral → granule synapses distal to the mitral soma, which were strengthened 3×, did not cause much self-inhibition, as the recurrent granule ─┤mitral synaptic strength decayed distally.

Each individual synaptic weight was finally set log-normally [89,90] with standard deviation 25% around its putative value.

#### Consistency of granule ─┤mitral decay with gating of spikes on lateral dendrites

Focal GABA uncaging on the mitral lateral dendrite did not block spike propagation in the rest of the dendrite and revealed reduction in granule ─┤mitral strength with distance [32]. We incorporated this reduction (sub-section above), and found analogously that inhibition on mitral spikes along a lateral dendrite by granule cells activated by these spikes, background mitral input, and a strongly-firing mitral cell midway along the dendrite, did not block spike propagation (Fig 2E). However, with inhibition from multiple mitral cells, gating of spikes might occur, analogous to that probed with wider activation [56] or removal [55] of inhibition, and modeled (without above granule ─┤mitral conductance reduction with distance) [49]. Thus, we expect that spike gating effects will not be strong for sparse activation of the bulb, but will emerge with broader activation.

#### Circuit level constraints

When we naively set synaptic weights based on evoked / spontaneous post synaptic potentials / currents, network effects like mean firing rates and activity dependent inhibition were quantitatively different from experiment. Therefore, we replicated various network level experiments, at each stage refining a subset of synaptic weights or connectivity, while maintaining previous results (Table 2). Final weights are in Table 1.

### What was simplified in the model, and why

1. Most input to mitral and PG cells, is via ET cells [24,91]. We expect this to cause further averaging and non-differential input to mitral and PG cells within a glomerulus. In addition, the Poisson spike train input that we provide could as well represent ET input rather than ORN input. This is justified as it has been observed that juxta-glomerular cells have response profiles similar to ORNs [92]. Thus we omitted ET cells from our current model. It would be interesting to study the roles of these ET cells, one of which could be to aggregate excitation from ORNs and inhibition from lateral glomeruli via short-axon cells.
2. The non-synaptic inhibitory action of PG cells on ORN terminals [93] was not included. As above, our Poisson spike train input represents the ‘processed’ output of ET cells. Intrinsic imaging reveals that glomerular responses are additive [94,95] (although at the mitral tuft, Ca^2+^ responses could be slightly hypo-additive, reaching only ~0.8 of the predicted sum on average [28]). Whether the inhibition of PG cells on ORN terminals aids or hinders linearity is an open question.
3. Various papers report long-lasting depolarizations and/or all-or-none mitral responses in slice to nerve shock [96–98] and *in vivo* at high odor concentrations [99]. However, others report short-duration and/or graded responses even to nerve shock *in vitro* [29,100]. *In vivo*, at concentrations of interest (1% or 2% saturated vapor), we expect graded responses, as corroborated by the very experiments we modeled [16–18]. It is possible that we have not modeled an excitatory pathway (say glutamate spillover) causing long-lasting depolarizations *in vitro*, that is quenched by inhibition *in vivo*, but that can be considered folded into our ORN spike trains and effective PG cell inhibition.
4. We have not modeled the inter-glomerular network formed by short-axon cells. Short axon cells have been reported to inhibit ET cells in glomeruli up to 600 μm away [101], and / or possibly excite PG cells [102], at multiple glomeruli [103]. Their effect on mitral cells is inhibitory *in vitro* [102,101]; yet very little inter-glomerular interactions were found in mitral tufts *in vivo* [28]. Also, short axon cells provide inhibition to mitral cells possibly from multiple glomeruli [24,102], averaged via ET or PG cells and distributed to multiple sister mitral cells, thus we don’t expect them to cause decorrelation between sister mitral cells. The general understanding in the field is also that SA cells along with ET cells perform a global bulbar normalization of activity [9,104], rather than cause differential responses between cells. Thus current published results imply a minimal role for SA cells in cross-glomerular effects such as decorrelation, and we have therefore not modeled them. However the anatomical connectivity of SA cells is highly suggestive of inter-glomerular effects, and this would be interesting to explore in a more complete model with further experimental input.
5. We have not modeled PG cell axons inhibiting nearby glomeruli, as they are rare but can extend over 4–5 glomeruli [105]. Also, the odor-activated glomeruli affecting a given mitral cell are sparse [71,4], hence far apart on average, and possibly not within reach of PG cell axons.
6. We also did not model gap junctions between M/T cells, between M/T and ET cells, and between M/T and PG cells in the glomerulus [106]. We expect gap junctions to perform further averaging in the tuft, possibly strengthening our results as in point 1) above.
7. Granule cells in our model were activated asynchronously since their leak reversal potentials were distributed normally (see ‘Network construction and connectivity’ sub-section above) leading to different spiking thresholds, and input synaptic strengths were distributed log-normally (see ‘Synaptic strengths’ sub-section above). Further, the synaptic delays of synapses from aggregated singly-connected granule cells to mitral cells were distributed exponentially to offset the effect of aggregation (see ‘Aggregation of non-shared granule cells sub-section above). Thus composite and long-lasting IPSPs in mitral cells, composed of multiple asynchronous IPSPs from multiple granule cells, are achieved in our simulations (S1 Fig) as observed [107,33,108,36,37]. A partial mechanism involving A-type Potassium current and its interplay with AMPA and NMDA receptors in the granule cell has been reported [108]. While our granule cell model did have A-type K current in the soma and dendrites, and AMPA and NMDA receptors with kinetics and ratio as experimentally observed (sub-sections above), we did not vary these as we obtained reasonably long composite IPSPs as above. Indeed granule cells appear to release neuro-transmitter causing unitary IPSPs in a mitral cell even hundreds of milliseconds after mitral excitation to the granule cells has stopped [107,33,108,36]. A recently reported mechanism of asynchronous synaptic release after ending of excitation, in the zebrafish, involved a calcium wave [109]. Thus, the full mechanism of asynchronous activation and neuro-transmitter release in granule cells is open for further modeling.
8. Tufted cells are similar to mitral cells, just smaller, and have not been modeled explicitly. For purposes of summation and lateral inhibition, we expect tufted cells to function similar to mitral cells. Though, recent studies indicate that tufted cells fire with lower latencies compared to mitral cells [110,111]. Mitral cell latencies approach those of tufted cells when bulbar inhibition is blocked [110], in particular when PG cell inhibition is blocked according to a preliminary report (Fukunaga, et al., Society for Neuroscience poster, 2012). This seems to suggest that PG cells differentially inhibit mitral cells compared to tufted cells and/or ORNs connect strongly to tufted cells and weakly to mitral cells [110]. Thus in the absence of connectivity data, we have currently not distinguished between tufted and mitral cells. Since the phase decorrelation study [18] that we simulated, compared nearby sister M/T cells (on the same octrode), we expect that they mostly compared mitral-mitral or tufted-tufted sister pairs. Thus, we do not expect that the phase decorrelation between sister cells is an artifact of the phase difference between tufted and mitral cells.
9. We also did not include the parvalbumin-expressing (PV) interneurons in the external plexiform layer, which form reciprocal dendrodentritic connections with mitral cells. These have been recently reported to take input from multiple mitral cells and are broadly tuned to odors, unlike granule cells which are more selective and narrowly tuned [112,113]. They seem to linearly control mitral output [112], thus possibly performing gain control. Since these are broadly tuned to odors, we do not expect them to affect differential inhibition, and they may be considered folded into the singly-connected granule cells receiving background input in our model.
10. We have also neglected Blanes cells that inhibit granule cells [114], other deep short-axon cells that inhibit PG cells and granule cells [115], cholinergic interneurons [116], and other interneurons in the bulb [27,117] due to their smaller number and lack of detailed information.
11. We also did not include centrifugal input to the bulb in our model, as we considered only anesthetized preparations where centrifugal input may not play as strong a role as in awake animals [77,78,118].
12. We did not include any learning in our model, again since we were focusing on recordings from anesthetized preparations, which have lower centrifugal input that is critical for learning [119]. We did not include adaptation in our model since the experimental protocol was designed to minimize adaptation. We tested short-term depression in the granule ─┤mitral synapse [120], and preliminary results indicated that the linearity as measured by pulse-trains degraded with strong depression.
13. We modeled only a small number of microcircuits, and only a small number of mitral cells within each, as we were computationally limited.
14. We combined experiments on rats and mice. Our model dimensions, cellular and synaptic numbers, and synaptic time constants were largely those of rat. Synaptic weights were fine-tuned from experiments on both rats and mice. We used an anesthetized respiration period of 0.5 s as for mice. Firing rates of mouse and rat mitral cells for air and odor were not too dissimilar (using data of [18] vs [16]), and bulbar circuitry is very similar, so we expect that our simulation results will match experiments across the two rodents.

### Stimulus protocols and analyses

Whenever replicating experiments, we broadly followed the analogous experimental protocol and analysis, in particular for activity dependent inhibition [37], linear kernel fitting and prediction [17], phase and delta-rate decorrelation [18], and odor morphs [16]. Data from other studies have been re-plotted from original data [16–18] or digitizing plot images [37]. The protocols and analyses are briefly described below and differences from the experiment noted.

#### Cellular electrophysiology

For action potential initiation in the mitral cell (Fig 2A–2C), we activated 33 of 400 ORN → mitral synapses on mitral cell A and 4 of 50 ORN → PG synapses on each PG cell over 4 ms for weak shock, and 66 of 400 ORN → mitral and 8 of 50 ORN → PG synapses for strong shock. In each case, the fraction of total synapses per cell activated for the mitral cell was the same as for the PG cells in the mitral cell’s glomerulus, representing the fraction of ORNs activated with electrical shock.

For action potential propagation in a mitral cell B (Fig 2D and 2E), we used the *default* network with B super-inhibiting A via extra granule cells having enhanced synaptic weights around A’s soma (Fig 4D, Table 1). Both mitral cells received 15 Hz input at the tuft, and granule cells received 35 Hz *in vivo* background [39] as proxy for network activity.

#### Activity dependent inhibition [37]

For activity dependent inhibition *in vitro* (Fig 3), we probed randomly connected mitral cells A and B, 50 μm apart, in the *slice* version (Table 3) of the default network (most mitral cells were randomly connected via granule cells based on proximity of dendrites). We injected current in mitral cell A at 250 ms after start of simulation for 400ms, and measured mean firing for the injection duration. We repeated the same with different current injections in A to obtain f-vs-I curve. These f-vs-I simulations were repeated with current injection of 1.2 nA in mitral cell B, started 5 ms before injection in A, and lasting till end of injection in A (Fig 3C). We repeated this ‘experiment’ for 10 network instances generated with different seeds, and selected 5 having the largest peak-reduction in firing rate at any current (since ~half of experimental pairs showed activity dependent inhibition [37]). The mean reduction in firing of A (due to injection in B) as a function of firing rate in A (no current injection in B) was calculated from the data of these 5 maximally inhibiting pairs (Fig 3D).

For activity dependent inhibition *in vivo* (Fig 6A, Fig 4E and 4F), we probed a super-inhibitory connection from B to A in the *default* network, or in modifications of the same (Fig 6B–6E). Receptor (ORN) input as Poisson spikes at a constant rate was provided instead of current injection. 35 Hz Poisson spikes were provided to granule cells as proxy for *in vivo* background. The firing rate in A was averaged over a 400 ms interval after a 100 ms settling time. We repeated the same with different rate inputs to A to obtain an f-vs-ORN-input curve. The f-vs-ORN-input curve was repeated with B receiving 5Hz ORN Poisson spikes and with B receiving 10 Hz to probe the inhibitory effect of B on A. These simulations were repeated for 10 network instances with A and B separated by 400 μm. The mean f-vs-ORN-input curves across these 10 instances, for each of the three inhibitory cases were plotted (Fig 6A–6E). We computed the point-wise difference between the mean uninhibited curve and the mean curve with 10 Hz input to B. The mean of these differences yielded a single number representing the mean reduction in A due to 10 Hz input to B, across inputs to A, and across network instances. This mean reduction in A due to B, as a function of their separation was calculated from simulations as above and plotted (Fig 4F). We interchanged A and B in the above simulations to obtain the mean reduction in B due to A as a function of their separation (Fig 4E).

#### ORN spike trains

We distributed 400 ORN → mitral synapses on each mitral tuft, randomly on the tuft compartments, consistent with observations [29]. We distributed 50 ORN → PG synapses, randomly on the two dendrites of each PG cell.

We did not model ORNs biophysically, rather as Poisson spike trains afferent on to above mitral and PG cell synapses. For simulating activity dependent inhibition *in vivo* (Fig 4D–4F), we gave Poisson spike trains of constant firing rate to mitral cells A and B. However, to represent odor / air inputs, the firing rate was time-varying and generated as below. This firing rate time-series for each glomerulus was used to generate different Poisson spike trains for each of the ORN → mitral and ORN → PG synapses in the glomerulus. We generated all Poisson spike trains with a 1 ms refractory period.

#### ORN kernels and firing rate time-series

For the ORNs of each glomerulus, we first generated linear kernels for air and two odors, as Gaussians in time with latency-to-peak *t*_*p*_ = 150 to 350 ms similar to mitral kernels [17], and slightly larger width *σ*_*p*_ (as mitral responses become sharper post shaping by inhibition) distributed uniformly over 250–450 ms. Each Gaussian was pre-multiplied by a factor to ensure it started from zero. Thus the excitatory kernel was

$$k\left(t\right)=k_{p}{\left(t/t_{p}\right)}^{t_{p}/2\;\sigma_{p}}\text{ exp}\left(-{\left(t-t_{p}\right)}^{2}/2\sigma^{2}\right)$$

The amplitude *k*_*p*_ of the air / odor kernel was uniformly distributed to get air / odor ORN peak firing rates from 0.8 / 2.4 Hz to 3 / 9 Hz, after convolution with respiration waveform, but before adding air and odor responses together (Fig 7C). These values were chosen to match experimental ORN air / odor firing rates [41,42]. We chose purely excitatory kernels for the model, so as to not mix up the lateral inhibitory effects with a direct inhibitory ORN component from the tuft. We did use excitatory- and inhibitory-component kernels i.e. a weighted difference of above Gaussians randomly displaced in time, for the simulations in Fig 5J. The simulations suggest that excitatory-inhibitory kernels produce more realistic i.e. sharper and more negative going responses compared to those from purely excitatory kernels as in Fig 5G.

We used the same set of ORN air and odor kernels for both the tracheotomized and the freely-breathing protocols, to compare the two in the same network instance. The set of air and odor ORN kernels along with the corresponding network instance was analogous to a distinct rat / mouse. For the tracheotomized and freely-breathing protocols (details in sections below), first an odor concentration time-series was generated as a random pulse-train or a respiratory waveform respectively. Similarly an air flow-rate time-series was generated. The concentration time-series was convolved with the odor kernel, while the air flow-rate time-series was convolved with the air kernel. Their sum along with a constant 0.5 Hz baseline gave the firing rate time-series of the ORNs of a given glomerulus (Figs 5D and 7C), from which the ORN spike trains were generated.

For most of the simulations we assumed that the ORN responses scaled linearly with odor concentration and air-flow. There is some evidence that ORN responses scale linearly with odor concentration roughly over 1 order of magnitude around 1% saturated vapor [121,122]. Glomerular responses might also summate over two odors [94,95,28]. There is also some evidence for ORN responses scaling with air flow rate at constant odor dilution [123]. However, we also tested the effect of a sharp non-linearity at the output of the ORNs (S6H Fig). For this, the ORN firing rate ORN_i_ in Hz calculated above was transformed to ORN_o_ in Hz, using a logistic function centered at 7.2 Hz with 1 Hz steepness:

$${\text{ORN}}_{\text{o}}=18/\left(1+\text{exp}\left(-\left({\text{ORN}}_{\text{i}}-7.2\right)\right)\right)$$

#### Linear kernel fitting and prediction (tracheotomized rat) [17] (Fig 5, S2 and S3) Figs

For the tracheotomized protocol (Fig 5D) analogous to experiment [17], we generated pseudo-random on-off pulses of odor as a binary-level, 7-bit, m-sequence, since it has a broad-band flat spectrum, following a previous study [124]. Each bit was used to turn a pulse of duration 50ms on/off. The full 7-bit sequence was of duration 350 ms. The pulse height corresponded to either 1% saturated vapor or 2%. The binary m-sequence was convolved with an exponentially decaying function of time constant 40 ms, which captured the experimental odor valve’s opening/closing. This convolution gave the true dynamical odor concentration in the air.

We then convolved this true concentration time-series with the ORN odor kernel to obtain the ORN firing rate waveform for this protocol. This odor m-sequence rode on a constant air/suction pedestal. The constant pedestal was convolved with the air kernel to get the air firing waveform which was added to that of the odor, along with a constant 0.5 Hz background.

The flow rate used in the experiment [17] for tracheotomized rats was 250 to 300ml/min, whereas peak flow rate for freely respiring rat is ~1000ml/min [125–127]. Thus, we set the pulse-on / air pedestal height for tracheotomized simulations at 1/3^rd^ the peak of the respiration waveform for freely breathing simulations. This also ensured that simulated firing rates of mitral cells remained in the physiological range as seen in the tracheotomized experiments [17], having already set them (Table 2) for freely-breathing experiments [16,18].

For every network instance, we ran 2 random pulse-train stimuli for each of two odors, and 1 two-odor overlapping random pulse-train stimulus (2 of the 4 single-odor pulse-train responses are shown in S2G and S2H Fig, and the binary-odor pulse-train response is shown in Fig 5G). We farmed 9 trials for each of these 5 random pulse-train stimuli on the cluster. We binned each central sister’s odor responses into 50 ms bins, averaged over the trials.

We fit the responses to the 2 random pulses stimuli per odor for each central sister, after subtracting a constant air background, with a 2 s long odor kernel discretized at 50 ms. We predicted each sister’s response to the sum of random pulses of two odors, using fitted kernels for the two odors. This analysis closely followed experiment [17] and $\sqrt{residual/noise}$ for the fits and predictions was calculated as defined there. Briefly, residual was the mean squared deviation of the fit / prediction from the averaged data, while the noise was the mean variance of the data, with the means over all time-bins.

#### Freely-breathing ORN input

For the freely-breathing protocols we replicated [16–18], we convolved the odor and air ORN kernels with a periodic half-rectified respiration waveform and summed the two with a 0.5 Hz baseline to generate the ORN firing rate in a glomerulus (Fig 7C). The respiration waveform was constructed as below (before half-rectification) to be similar to that in Fig 1C of a previous experiment [122].

resp(t)=0.6 dualexp(t, 0.06 T, 0.05 T)+0.5 dualexp(t, 0.3 T, 0.1 T)−0.1 dualexp((T−t), 0.03 T, 0.025 T) where dualexp(*t*,*τ*_1_,*τ*_2_) = (exp(−*t*/*τ*_1_) – exp(−*t*/*τ*_2_))/(*τ*_1_–*τ*_2_), and the respiration period *T* = 0.5 *s*. The respiration waveform was half-rectified assuming that the expiration flow is not odorous. However, expired air might carry back a reduced odor concentration (Gupta and Bhalla, personal communication). We scaled the odor response to correspond to a desired experimental concentration before adding it to the air response. In these experiments [16–18], anaesthetized mice breathed quite regularly at ~2 Hz and rats at ~1 Hz, hence we used periodic respiration, thus phase is the same as periodic time. We chose the respiratory period as 0.5 s since our simulations for freely-breathing responses were primarily to replicate the results in mice [18]. To avoid multiple simulations, we used these same responses to demonstrate linear prediction [17] and summation of freely breathing responses [16] in rats too.

We had set the glomerular input and inhibitory synaptic strengths (Tables 1 and 2), so that the default 1× scaling of the ORN odor response gave a mean mitral firing rate of 14 Hz for freely-breathing simulations. This was to match the experimental mean of ~10–15Hz for odor responses at 1% saturated vapor pressure (from data of replicated experiments [16,18]). For odorless air, the mean rate was 8 Hz, similar to the same experiments.

#### Phase-decorrelation (freely-breathing mouse) [18] (Figs 7 and 8)

For air and for each odor in every network instance, we ran 8 trials in parallel on the cluster with each trial having 2 respiratory cycles. We averaged over the second cycles of the trials and binned the average cycle response into 5 bins. Since typical mitral kernels had duration between 0.5 to 1 s [17] which were roughly twice the respiration period of 0.5 s, the first and second cycle responses were different, but later cycle responses were checked to be similar in our simulations. In the experimental analysis, a continuous 5 s or 10 s period was broken into cycles and averaged over. So the effect of the first cycle was negligible. Also, the periodic response has been seen to be stereotypical in shape neglecting the first cycle [16]. Thus, we are justified in averaging only the second cycles farmed in parallel.

As in the experimental analysis, we calculated the Pearson correlation of the mean single-cycle odor response of one central sister versus the other. We did the same for the air responses.

We calculated the delta-rate for each odor i.e. odor mean—air mean, with the mean over the bins of all the second cycles. We strung together the delta-rates for every odor in every network instance of one central sister into one vector, and for the other central sister into another. The Pearson correlation between these two vectors gave us the delta-rate correlation which measures how much the sisters co-vary in their mean rate responses to odor, compared to their air baselines.

#### Predicting freely-breathing response from tracheotomized response kernel (S4 Fig)

Since we used the same input ORN kernels to generate the freely-breathing and tracheotomized stimuli, we could use the mitral odor kernel fitted from the tracheotomized protocol to see how well it predicted the freely-breathing response. We convolved the fitted mitral odor kernel with the rectified respiration waveform / time-series and compared this prediction, using the $\sqrt{residual/noise}$ measure, to the mean simulated freely-breathing mitral odor response, after subtracting the mean simulated mitral air response (S4 Fig).

#### Odor morph fitting (freely-breathing rat) [16] (S5 Fig)

Following the replicated experiment [16], we simulated each central sister’s responses to odorless air *R*_*air*_(*t*), odor A *R*_*A*_(*t*), odor B *R*_*B*_(*t*), and 4 mixtures / morphs between A and B *R*_*AB*_(*t*) given by concentrations *C*_*A*_ and *C*_*B*_. These responses were fit using parameters: three free-form functions *F*_*air*_(*t*),*F*_*A*_(*t*),*F*_*B*_(*t*) corresponding to air and pure odor representations, a saturation frequency *r*_*max*_, and 8 weights *w*_*A*_(*C*_*A*_), *w*_*B*_(*C*_*B*_) monotonic with concentration, using equations below:

$$\begin{array}{c} R_{air}\left(t\right)\equiv R_{C_{A}=0,C_{B}=0}\left(t\right)=r_{max}\;f\left(F_{air}\left(t\right)\right)\\ R_{A}\left(t\right)\equiv R_{C_{A}=1,C_{B}=0}\left(t\right)=r_{max}\;f\left(F_{A}\left(t\right)\right)\\ R_{B}\left(t\right)\equiv R_{C_{A}=0,C_{B}=1}\left(t\right)=r_{max\;}f\left(F_{B}\left(t\right)\right)\\ R_{AB}\left(t\right)\equiv R_{C_{A},C_{B}}\left(t\right)=r_{max}\;f\left(w_{A}\left(C_{A}\right)\;F_{A}\left(t\right)\;+\;w_{B}\left(C_{B}\right)\;F_{B}\left(t\right)\;+\;F_{air}\left(t\right)\right) \end{array}$$

where *f*(*x*) = exp(4.39*x*)/(1+exp(4.39*x*)) was a sigmoidal output non-linearity. Measured responses *R*_*air*_(*t*), *R*_*A*_(*t*), and *R*_*B*_(*t*) could not be equated with ‘internal representations’ *F*_*air*_(*t*), *F*_*A*_(*t*), and *F*_*B*_(*t*), since the former are rectified, while the latter may be partially negative.

We also tested a model similar to the pulse-trains experiment [17], where the weights were fixed at the normalized concentrations, and a simple rectifier was used instead of a sigmoid at the output.

In the experimental analysis [16], 7 s of odor/air response were extracted corresponding to ~7–8 respiratory cycles (period ~1 s for rat), after leaving out the first cycle. The average single-cycle 1 s response was computed by averaging over these cycles and binning into 17 bins. In the simulation analysis, we reused the freely-breathing air / odor responses data, from phase-decorrelation simulations above, for the mouse having a respiratory period 0.5 s. The binsize was maintained by binning the 0.5 s period into 9 bins. We expect that these differences will not affect the results qualitatively. We used $\sqrt{residual/noise}$ for odor morph fits also to compare experiment and simulations.

#### Scaled pulses (S6 and S7 Figs)

To compare responses across a larger concentration scale, we used 200 ms pulses of odor scaled to 0%, 1/3%, 2/3%, 1%, 2% and 5% saturated vapor. The protocol was the same as the tracheotomized protocol (Fig 5D), except that the random pulses were replaced by above single pulse. 9 trials were simulated for each scaled pulse input for each of 50 *default* network instances.

For S6A Fig, we first computed normalized-latency-to-spike after odor onset averaged across these 9 trials, for each of 2 mitral cells in the 50 instances, for each scaling / concentration. We normalized for different odor input latencies in each network instance, by dividing by the mean latency to spike for each mitral cell across scaled pulses. We finally plotted the mean normalized-latency-to-spike across the 100 mitral cells, as a function of scaling / concentration.

For S6B and S7 Figs, we binned each scaled odor response to form a firing rate time histogram of length 1.7 s and bin size 50 ms, following odor onset, and averaged it over 9 trials. We also subtracted the mean air response from each scaled odor response. For S6B Fig, we computed the mean normalized-latency-to-peak of the time histogram after odor onset, as for the latency to spike.

For S7 Fig, we correlated each scaled odor response against the 1% odor response for every network instance, yielding a distribution of correlations whose mean and standard deviation are plotted versus the input concentration. Similarly, for peaks of scaled responses, the mean and standard deviation of their distribution across all network instances are plotted versus the input concentration. The above was repeated with various circuit components removed to probe their effects on linearity.

## Supporting Information

S1 Fig Lateral versus recurrent inhibition, and composite long-lasting inhibition. **B,D** are voltage traces of mitral cell A (red), while **C,E** are those of mitral cell B (green) in schematic **A**. **A.** Schematic for the simulations (same as Fig 3**A**). **B-C.** Voltage responses of cell A (red) and cell B (green) respectively, with 750 pA injected in cell A for 400 ms, and 2000 pA injected in cell B for 405 ms (starting 5 ms earlier). Inset in **B** shows that the long-lasting inhibitory potential is composed of multiple, smaller time-scale, asynchronous inhibitory post-synaptic potentials from multiple granule cells as observed experimentally. **D-E.** Same as **B-C** but without any current injection in cell B. Firing rate of cell A in panel **B** is lower than in panel **D**, due to lateral inhibition from cell B. This figure is similar to Fig 2B in [37]. Note that action potentials in our model mitral cell have a small undershoot during the hyperpolarization phase, which though not seen in [37] is seen in Fig 3 of [128].
(TIF)

S2 Fig Experiment and model fits of random on-off pulse-trains with linear kernels. **A-B:** Experiment [17]: Kernels for two odors (red and blue) obtained by fitting mitral cell responses to pulse-trains in **C-D**. **C-D.** Experiment: Mean mitral responses (gray) with SEM bands (12 trials), to random on-off pulse-trains for two odors (background bars in magenta and cyan), along with their linear fits (red and blue). **E-H:** Model: As for **A-D** in a *default* network instance (9 trials). The model input ORN kernels had only an excitatory component (Materials and Methods), hence the model mitral responses are not as sharp and negative-going as the experiment. With input ORN kernels having excitatory and inhibitory components, responses were more realistic, but we chose purely excitatory kernels to not confound inhibition from interneurons. However, linearity was maintained even with dual-component ORN kernels (Fig 5J).
(TIF)

S3 FigPredicted effects of concentration on linearity.Goodness of fits and predictions, and kernels of simulated responses to 2% saturated vapor random pulse-trains compared with 1%: A-B. Distributions of $\sqrt{residual/noise}$ for **A.** fits of mitral cell responses to single odor random pulse-trains, and **B.** predictions of responses to two odor random pulse-trains, for ORN input corresponding to 1% saturated vapor (100 mitral cells in 50 network instances). Fits / predictions with $\sqrt{residual/noise}\;<\;1$ are acceptable. **C-D.** As for **A-B** but for input corresponding to 2% saturated vapor pressure (60 mitral cells in 30 network instances). **E.** Histogram of Pearson correlations between mitral kernels fitted to 1% versus 2% saturated vapor responses (120 odor kernels for two different odors to each of 60 mitral cells in 30 different network instances). **F**. Control histogram of Pearson correlations between independent odor kernels at the same concentration (100 pairs of mitral odor kernels at 1% saturated vapor and 60 pairs at 2% saturated vapor). (There is a bias towards positive correlation since ORN kernels were purely excitatory.)Correlations between mitral kernels at 2% saturated vapor versus those at 1%, for the same odor and in the same network, were only slightly higher than control correlations between kernels for independent odors at the same concentration. Thus the kernels across 2% and 1% saturated vapor were not similar, even though fits and predictions at 2% and 1% saturated vapor were acceptable, suggesting that linearity is limited across concentration.(TIF)

S4 FigRespiratory predictions from linear kernels.
**Model: A-B.** Mean mitral cell responses (to the second respiratory cycle input in Fig 7C), in a *default* network instance, to two odors (magenta and cyan) with SEM bands (8 trials) after subtracting the mean air response; along with corresponding predictions (red and blue) using kernels obtained from random pulse-train fits. **C.** Distribution of $\sqrt{residual/noise}$ for predictions of mitral respiratory responses using kernels obtained from fitting random pulse-trains (200 odor responses to two odors for 100 mitral cells in 50 network instances). SD not SEM was used to calculate noise. Predictions with $\sqrt{residual/noise}\;<\;1$ are acceptable. **Experiment: D.** As for **C.** re-plotted from experimental data (Gupta and Bhalla, personal communication) (23 odor responses predicted using respiratory waveform comprising full inhalation, with rectified half-exhalation as that gave the best predictions compared to no exhalation or rectified full-exhalation.)(TIF)

S5 FigLinear fits to odor morphs.Khan, et al. [16] measured mitral responses to odor morphs i.e. binary odor mixtures at concentrations (C_A_,C_B_) = (0,0), (0,1), (0.2,0.8), (0.4,0.6), (0.6,0.4), (0.8,0.2), and (1,0) % saturated vapor [16]. They were able to fit these with just scaled summations of pure odor and pure air representations (Materials and Methods).
**Experiment** [16]**: A-C.** The weights to scale the pure representations to fit each morph were also free parameters, and a sigmoidal non-linearity at the output was present, as in the original analysis [16]. **A.** Example mean responses of a mitral cell to two odors and air (solid red, blue, and black) with SEM bands (36 trials); and their fits / ‘internal representations’ (dashed magenta, cyan, and gray). Morph / mixture responses and their fits are not shown to avoid clutter. **B.** Fitted scaling / weights that multiplied the pure representations in fitting the morphs (dashed magenta and cyan) are compared to linear weights (red and blue). **C.** Distribution of $\sqrt{residual/noise}$ for the fits (38 mitral-morph combinations). SD not SEM was used to calculate noise. Fits with $\sqrt{residual/noise}\;<\;1$ are acceptable. **D-F.** As in **A-C**, except the weights were set to the normalized concentrations i.e. were linear, and a simple rectifier output was used, similar to the random pulses fitting [17]. **Model: G-L.** Analogous simulations to **A-F**, except 8 trials for mean and SEM in **G,J** and 100 mitral-morph combinations in **I,L**.(TIF)

S6 Fig Latencies to spike and peak drop with concentration.A-B. Mitral cell responses to 200 ms long odor pulse input, scaled at 1/3, 2/3, 1, 2, and 5% saturated vapor pressure, were simulated in 50 instances of the *default* network. **A.** Mean normalized-latency-to-spike after odor onset versus ORN input concentration, for 100 mitral cells (2 in each *default* network instance). Standard error is indicated by the error bars. Latency was normalized to the mean latency for each mitral cell across the scaled responses, since each network instance received ORN input having different latencies. The amplitudes of the air and odor responses were comparable for low concentrations; hence their times to spike were similar. But for higher concentrations, the times-to-spike dropped. **B.** As in **A**, but for the mean normalized-latency-to-peak of the firing rate response (binned at 50ms, air response was subtracted) after odor onset. ORN kernels used to generate this input had an only excitatory component. We expect more complex behavior with ORN kernels having excitatory and inhibitory components.
(TIF)

S7 Fig Non-linearity in scaling of responses to odor pulses of wider-ranging concentrations.A-B. Example simulated responses to scaled odor pulse input in *default* network after subtracting the air response: **A.** Responses of a central mitral cell to scaled odor pulses of 200 ms duration (red bar at the start of the responses), scaled to 1/3 (red), 2/3 (blue), 1 (green), 2 (magenta), and 5 (cyan) % saturated vapor pressure. **B.** Pearson correlation of each scaled response to the 1% response as a function of input concentration. A Pearson correlation close to 1 signifies that the shape / time profile of the response is unchanged compared to the 1% response. **C.** Peak of each scaled response versus input concentration. **D-H.** Analysis of responses (minus air response) to scaled odor pulse inputs, created from different odor/air kernels, in 5 different networks with a range of modifications to the *default*. 50 runs were carried out for random instances of each class of network: **D.** *default*, **E.** *default* without lateral mitral cells, **F.** *default* without recurrent (no singly-connected granule cells) and feed-forward (no PGs) inhibition, **G.** *default* without any interneurons, **H.** *default* with sigmoidal saturating input.Top row shows schematic of network. Middle row shows mean and standard deviation of the distribution of Pearson correlations between the scaled response and the 1% response, as a function of the input concentration. Standard deviation of the distribution has been shown as an ‘error bar’, but is over different input kernels and networks. A Pearson correlation co-efficient close to 1 implies that the scaled response is similar to the 1% response. Bottom row shows the mean and SD of the distribution of response peaks as a function of input concentration. As in middle row, ‘error bars’ refer to standard deviation across networks and kernels. There is a saturating trend to the response peak with concentration. Odor responses (after subtracting the air response) in **F** (with lateral inhibition) are larger than in **G** (without inhibition i.e. no interneurons), since air responses are smaller for **F**. The combined air + odor responses are larger for **G** (no inhibition).Even in the *default* network, the shape and the peak scaling of responses saturates with concentration. Removing self-inhibition **F**, or using non-linear inputs **G** degrades both even further.
(TIF)

S1 Video Visualization of phase-decorrelation and network activity in the olfactory bulb model. (MP4)
