# Supplementary figures and images for: Bulbar Microcircuit Model Predicts Connectivity and Roles of Interneurons in Odor Coding
Source: PLoS One. 2015 May 5;10(5):e0098045. doi: 10.1371/journal.pone.0098045 (PMC4420273; doi:10.1371/journal.pone.0098045)

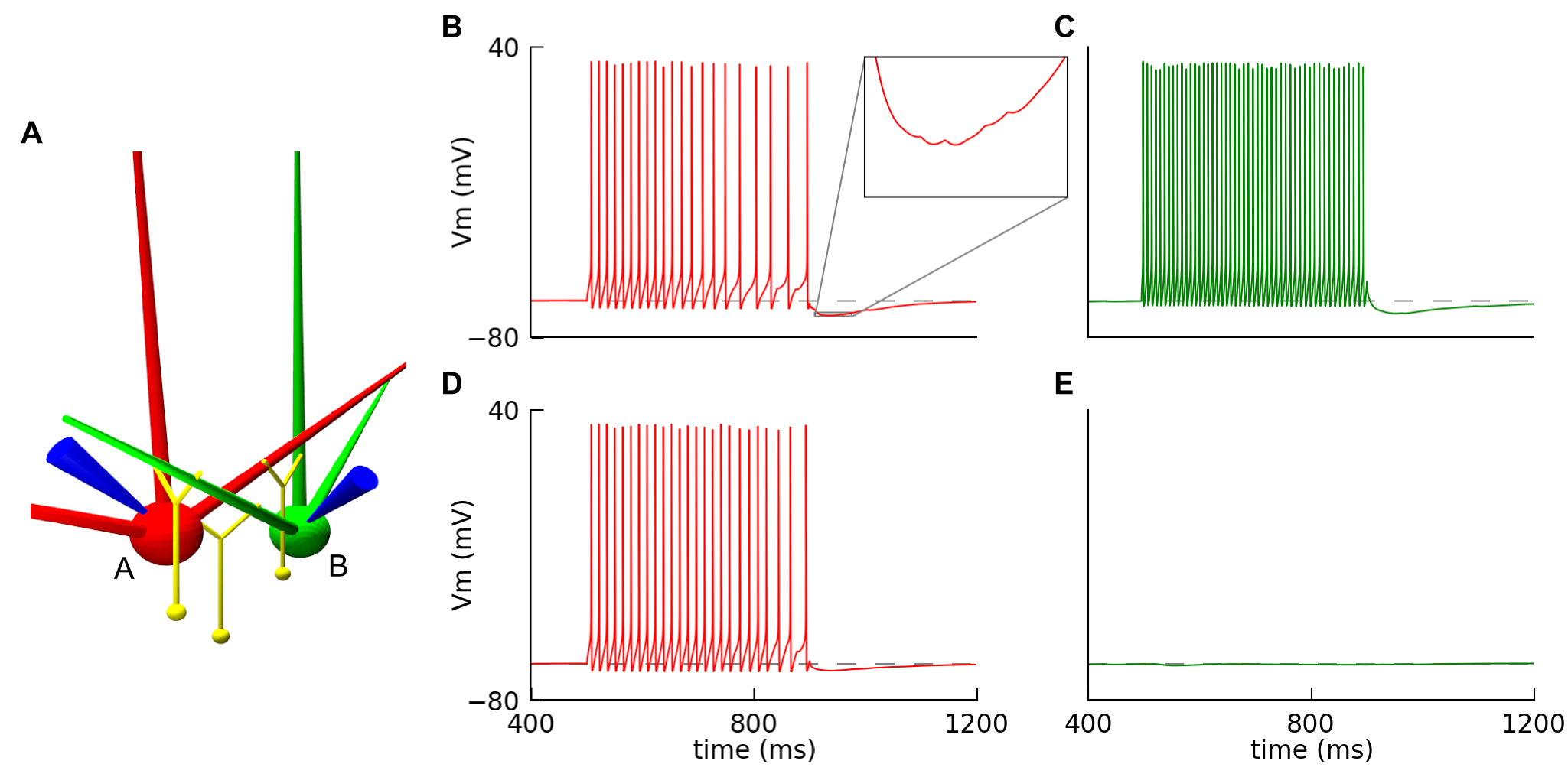

Supplement: S1 Fig — B,D are voltage traces of mitral cell A (red), while C,E are those of mitral cell B (green) in schematic A. A. Schematic for the simulations (same as Fig 3A ). B-C. Voltage responses of cell A (red) and cell B (green) respectively, with 750 pA injected in cell A for 400 ms, and 2000 pA injected in cell B for 405 ms (starting 5 ms earlier). Inset in B shows that the long-lasting inhibitory potential is composed of multiple, smaller time-scale, asynchronous inhibitory post-synaptic potentials from multiple granule cells as observed experimentally. D-E. Same as B-C but without any current injection in cell B. Firing rate of cell A in panel B is lower than in panel D, due to lateral inhibition from cell B. This figure is similar to Fig 2B in [37]. Note that action potentials in our model mitral cell have a small undershoot during the hyperpolarization phase, which though not seen in [37] is seen in Fig 3 of [128]. (TIF) [file pone.0098045.s001.tif]

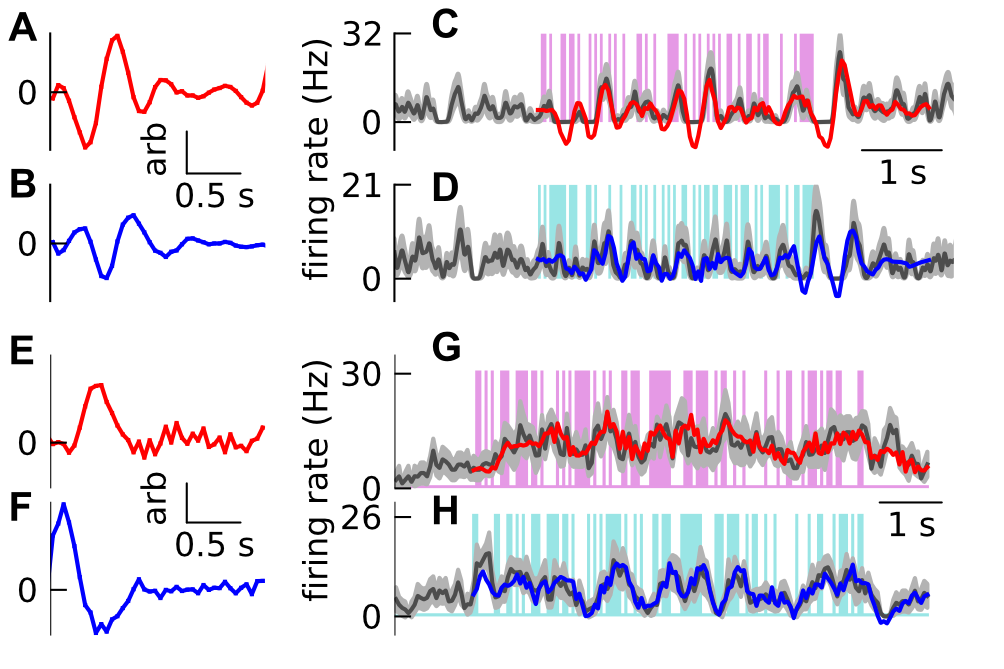

Supplement: S2 Fig — A-B: Experiment [17]: Kernels for two odors (red and blue) obtained by fitting mitral cell responses to pulse-trains in C-D. C-D. Experiment: Mean mitral responses (gray) with SEM bands (12 trials), to random on-off pulse-trains for two odors (background bars in magenta and cyan), along with their linear fits (red and blue). E-H: Model: As for A-D in a default network instance (9 trials). The model input ORN kernels had only an excitatory component (Materials and Methods), hence the model mitral responses are not as sharp and negative-going as the experiment. With input ORN kernels having excitatory and inhibitory components, responses were more realistic, but we chose purely excitatory kernels to not confound inhibition from interneurons. However, linearity was maintained even with dual-component ORN kernels (Fig 5J). (TIF) [file pone.0098045.s002.tif]

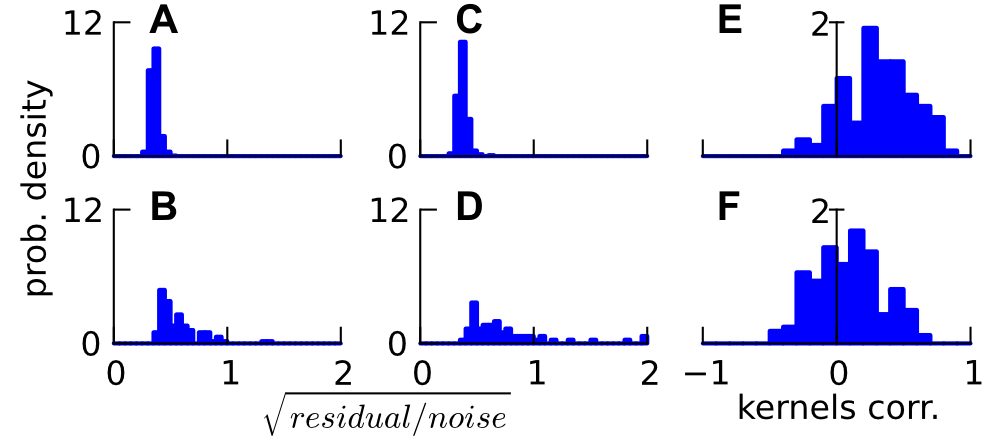

Supplement: S3 Fig — Goodness of fits and predictions, and kernels of simulated responses to 2% saturated vapor random pulse-trains compared with 1%: A-B. Distributions of residual/noise for A. fits of mitral cell responses to single odor random pulse-trains, and B. predictions of responses to two odor random pulse-trains, for ORN input corresponding to 1% saturated vapor (100 mitral cells in 50 network instances). Fits / predictions with residual/noise < 1 are acceptable. C-D. As for A-B but for input corresponding to 2% saturated vapor pressure (60 mitral cells in 30 network instances). E. Histogram of Pearson correlations between mitral kernels fitted to 1% versus 2% saturated vapor responses (120 odor kernels for two different odors to each of 60 mitral cells in 30 different network instances). F. Control histogram of Pearson correlations between independent odor kernels at the same concentration (100 pairs of mitral odor kernels at 1% saturated vapor and 60 pairs at 2% saturated vapor). (There is a bias towards positive correlation since ORN kernels were purely excitatory.)Correlations between mitral kernels at 2% saturated vapor versus those at 1%, for the same odor and in the same network, were only slightly higher than control correlations between kernels for independent odors at the same concentration. Thus the kernels across 2% and 1% saturated vapor were not similar, even though fits and predictions at 2% and 1% saturated vapor were acceptable, suggesting that linearity is limited across concentration. (TIF) [file pone.0098045.s003.tif]

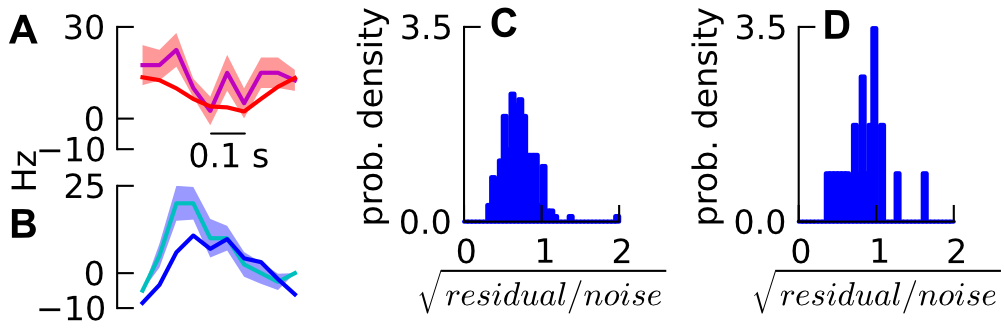

Supplement: S4 Fig — Model: A-B. Mean mitral cell responses (to the second respiratory cycle input in Fig 7C), in a default network instance, to two odors (magenta and cyan) with SEM bands (8 trials) after subtracting the mean air response; along with corresponding predictions (red and blue) using kernels obtained from random pulse-train fits. C. Distribution of residual/noise for predictions of mitral respiratory responses using kernels obtained from fitting random pulse-trains (200 odor responses to two odors for 100 mitral cells in 50 network instances). SD not SEM was used to calculate noise. Predictions with residual/noise < 1 are acceptable. Experiment: D. As for C. re-plotted from experimental data (Gupta and Bhalla, personal communication) (23 odor responses predicted using respiratory waveform comprising full inhalation, with rectified half-exhalation as that gave the best predictions compared to no exhalation or rectified full-exhalation.) (TIF) [file pone.0098045.s004.tif]

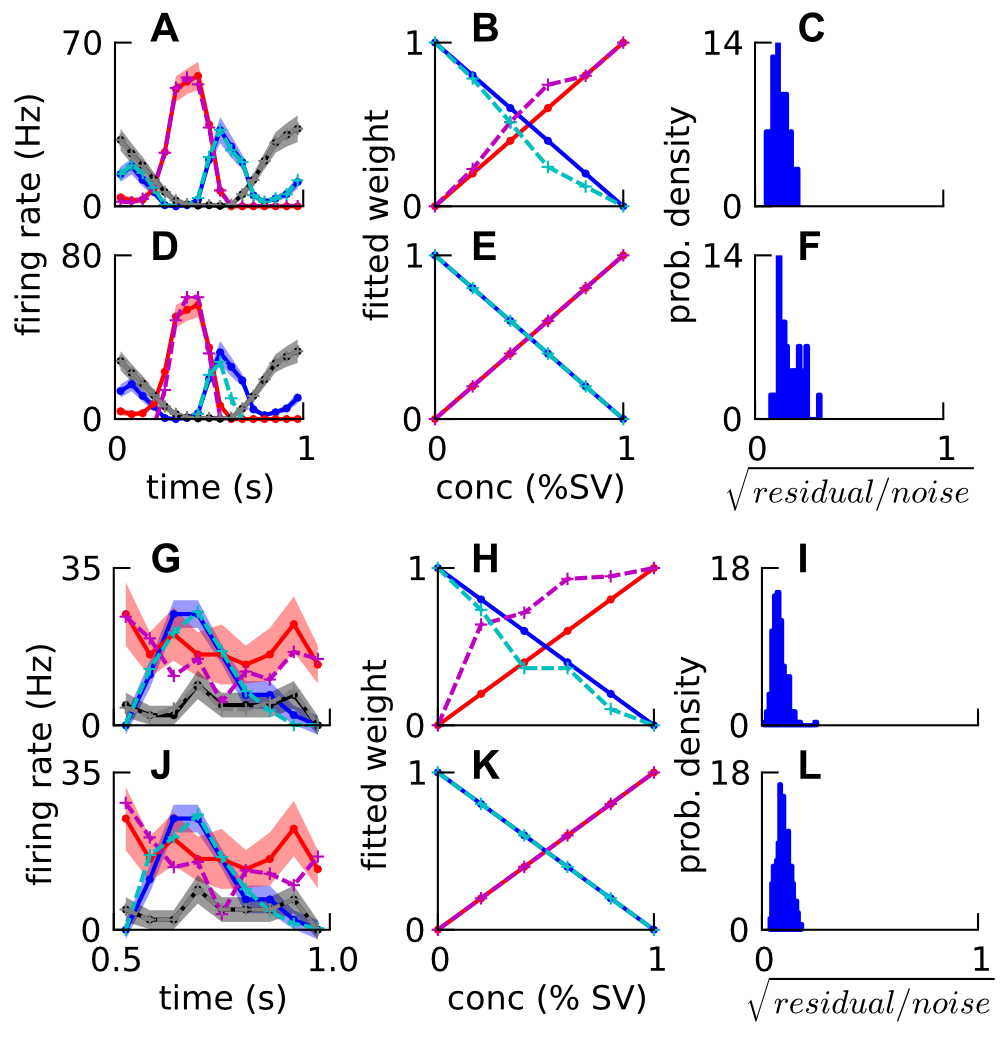

Supplement: S5 Fig — Experiment [16]: A-C. The weights to scale the pure representations to fit each morph were also free parameters, and a sigmoidal non-linearity at the output was present, as in the original analysis [16]. A. Example mean responses of a mitral cell to two odors and air (solid red, blue, and black) with SEM bands (36 trials); and their fits / ‘internal representations’ (dashed magenta, cyan, and gray). Morph / mixture responses and their fits are not shown to avoid clutter. B. Fitted scaling / weights that multiplied the pure representations in fitting the morphs (dashed magenta and cyan) are compared to linear weights (red and blue). C. Distribution of residual/noise for the fits (38 mitral-morph combinations). SD not SEM was used to calculate noise. Fits with residual/noise < 1 are acceptable. D-F. As in A-C, except the weights were set to the normalized concentrations i.e. were linear, and a simple rectifier output was used, similar to the random pulses fitting [17]. Model: G-L. Analogous simulations to A-F, except 8 trials for mean and SEM in G,J and 100 mitral-morph combinations in I,L. (TIF) [file pone.0098045.s005.tif]

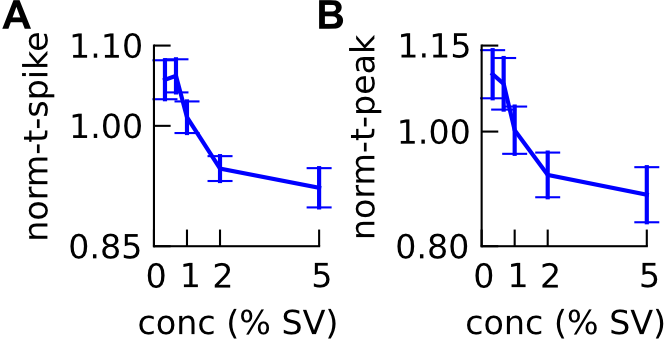

Supplement: S6 Fig — Mitral cell responses to 200 ms long odor pulse input, scaled at 1/3, 2/3, 1, 2, and 5% saturated vapor pressure, were simulated in 50 instances of the default network. A. Mean normalized-latency-to-spike after odor onset versus ORN input concentration, for 100 mitral cells (2 in each default network instance). Standard error is indicated by the error bars. Latency was normalized to the mean latency for each mitral cell across the scaled responses, since each network instance received ORN input having different latencies. The amplitudes of the air and odor responses were comparable for low concentrations; hence their times to spike were similar. But for higher concentrations, the times-to-spike dropped. B. As in A, but for the mean normalized-latency-to-peak of the firing rate response (binned at 50ms, air response was subtracted) after odor onset. ORN kernels used to generate this input had an only excitatory component. We expect more complex behavior with ORN kernels having excitatory and inhibitory components. (TIF) [file pone.0098045.s006.tif]

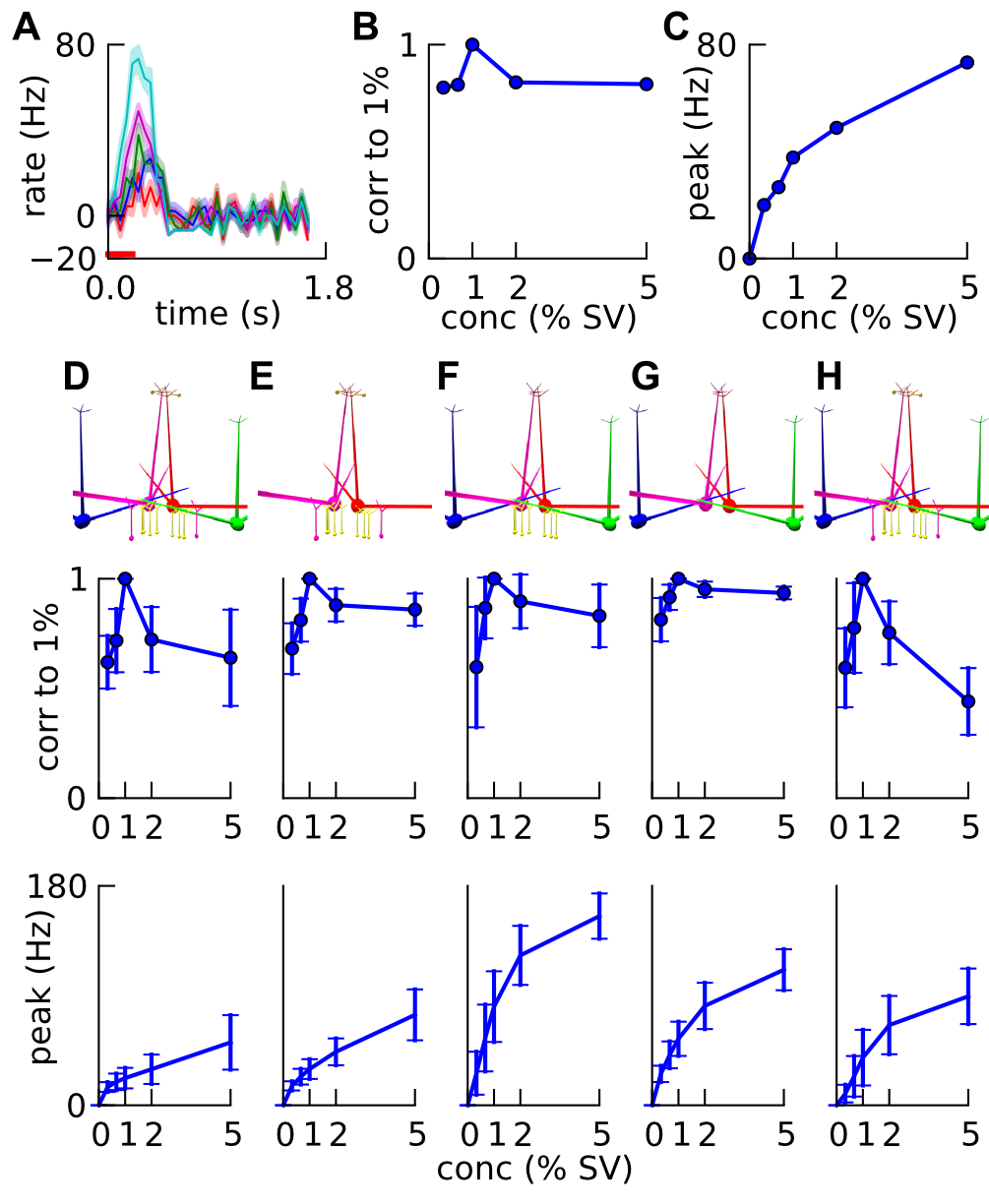

Supplement: S7 Fig — Example simulated responses to scaled odor pulse input in default network after subtracting the air response: A. Responses of a central mitral cell to scaled odor pulses of 200 ms duration (red bar at the start of the responses), scaled to 1/3 (red), 2/3 (blue), 1 (green), 2 (magenta), and 5 (cyan) % saturated vapor pressure. B. Pearson correlation of each scaled response to the 1% response as a function of input concentration. A Pearson correlation close to 1 signifies that the shape / time profile of the response is unchanged compared to the 1% response. C. Peak of each scaled response versus input concentration. D-H. Analysis of responses (minus air response) to scaled odor pulse inputs, created from different odor/air kernels, in 5 different networks with a range of modifications to the default. 50 runs were carried out for random instances of each class of network: D. default, E. default without lateral mitral cells, F. default without recurrent (no singly-connected granule cells) and feed-forward (no PGs) inhibition, G. default without any interneurons, H. default with sigmoidal saturating input.Top row shows schematic of network. Middle row shows mean and standard deviation of the distribution of Pearson correlations between the scaled response and the 1% response, as a function of the input concentration. Standard deviation of the distribution has been shown as an ‘error bar’, but is over different input kernels and networks. A Pearson correlation co-efficient close to 1 implies that the scaled response is similar to the 1% response. Bottom row shows the mean and SD of the distribution of response peaks as a function of input concentration. As in middle row, ‘error bars’ refer to standard deviation across networks and kernels. There is a saturating trend to the response peak with concentration. Odor responses (after subtracting the air response) in F (with lateral inhibition) are larger than in G (without inhibition i.e. no interneurons), since air responses [file pone.0098045.s007.tif]
